# Porous g‐C_3_N_4_ and MXene Dual‐Confined FeOOH Quantum Dots for Superior Energy Storage in an Ionic Liquid

**DOI:** 10.1002/advs.201901975

**Published:** 2019-11-27

**Authors:** Minjie Shi, Peng Xiao, Junwei Lang, Chao Yan, Xingbin Yan

**Affiliations:** ^1^ School of Materials Science and Engineering Jiangsu University of Science and Technology Zhenjiang 212003 P. R. China; ^2^ State Grid Jiangsu Electric Power Co., Ltd. Research Institute Nanjing 210000 P. R. China; ^3^ Laboratory of Clean Energy Chemistry and Materials State Key Laboratory of Solid Lubrication Lanzhou Institute of Chemical Physics Chinese Academy of Sciences Lanzhou 730000 P. R. China

**Keywords:** dual confinement, flexible supercapacitors, ionic liquids, pseudocapacitive behavior, quantum dots

## Abstract

Owing to their unique nanosize effect and surface effect, pseudocapacitive quantum dots (QDs) hold considerable potential for high‐efficiency supercapacitors (SCs). However, their pseudocapacitive behavior is exploited in aqueous electrolytes with narrow potential windows, thereby leading to a low energy density of the SCs. Here, a film electrode based on dual‐confined FeOOH QDs (FQDs) with superior pseudocapacitive behavior in a high‐voltage ionic liquid (IL) electrolyte is put forward. In such a film electrode, FQDs are steadily dual‐confined in a 2D heterogeneous nanospace supported by graphite carbon nitride (g‐C_3_N_4_) and Ti‐MXene (Ti_3_C_2_). Probing of potential‐driven ion accumulation elucidates that strong adsorption occurs between the IL cation and the electrode surface with abundant active sites, providing sufficient redox reaction of FQDs in the film electrode. Furthermore, porous g‐C_3_N_4_ and conductive Ti_3_C_2_ act as ion‐accessible channels and charge‐transfer pathways, respectively, endowing the FQDs‐based film electrode with favorable electrochemical kinetics in the IL electrolyte. A high‐voltage flexible SC (FSC) based on an ionogel electrolyte is fabricated, exhibiting a high energy density (77.12 mWh cm^−3^), a high power density, a remarkable rate capability, and long‐term durability. Such an FSC can also be charged by harvesting sustainable energy and can effectively power various wearable and portable electronics.

## Graphical abstract

A unique electrode based on dual‐confined FeOOH quantum dots (FQDs) is proposed, in which FQDs are confined in a 2D heterogeneous nanospace supported by g‐C_3_N_4_ and Ti_3_C_2_. Such an electrode exhibits superior energy‐storage behavior in a high‐voltage ionic liquid electrolyte, introducing a new avenue for breaking the bottleneck of the low energy density of quantum‐dot‐based supercapacitors.

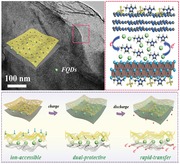

## 1 Introduction

Quantum dots (QDs) are a class of 0D nanomaterials with a diameter between 1 and 10 nm, which have attracted significant interest in the field of energy‐storage applications.^1^, ^2^, ^3^, ^4^ Owing to the unique nanosize effect and surface effect, pseudocapacitive QDs exhibit a high surface‐to‐volume ratio, a large number of active edge sites per unit mass, and fast and reversible redox reactions in high‐efficiency supercapacitors (SCs).^4^, ^5^, ^6^ In recent years, many efforts have been dedicated to achieving pseudocapacitive QDs by reducing the size of metal oxides/hydroxides (NiO, ZnO, MnO_2_, Nb_2_O_5_, Co(OH)_2_, etc.) to the quantum scale.^5^, ^6^, ^7^, ^8^, ^9^ These as‐obtained QDs have dominantly shown a rapid redox reaction along with a high pseudocapacitance close to the theoretical value.^8^, ^9^, ^10^ However, such pseudocapacitive behavior is mainly exploited in aqueous electrolytes. Since the energy density of SCs is proportional to the square of the operating potential window, attaining a high energy density of pseudocapacitive QDs‐based SCs using aqueous electrolytes, which is limited by the low decomposition voltage of water of ≈1.23 V, seems to be a dilemma.^11^, ^12^, ^13^

Ionic liquids (ILs), a class of room‐temperature molten salts, have been recognized as a promising candidate to substitute the aqueous electrolyte of SCs because of their intriguing properties, such as a wide operating voltage (>2.5 V), a low vapor pressure, nonflammability, and high thermal stability.^14^, ^15^, ^16^, ^17^, ^18^ Currently, extensive research has focused on the development of SCs using IL electrolytes, especially carbon‐based electrical double‐layer capacitors.^19^, ^20^, ^21^ Compared with the double‐layer capacitance of carbon electrodes, the pseudocapacitance resulting from reversible redox reactions of pseudocapacitive electrodes can deliver a much higher specific capacitance for SCs. However, although pseudocapacitive QDs have been demonstrated to possess large specific capacitance in aqueous electrolytes by virtue of redox reactions, reports of pseudocapacitive QDs‐IL electrolyte systems are relatively scarce to date,^22^, ^23^, ^24^ which is ascribed to the lack of suitable metal oxides/hydroxides presenting obvious pseudocapacitive behavior in IL electrolytes.^25^, ^26^ In addition, due to the high surface energy and van der Waals force of pseudocapacitive QDs, their intrinsic aggregation slows down the electron charge transfer and thus affects the electrochemical performance in IL electrolytes. This issue seriously hinders the implementation of pseudocapacitive QDs as high‐performance electrodes in IL electrolytes.

An effective approach is to confine pseudocapacitive QDs in a suitable nanospace to improve their structural stability as well as their electrochemical properties.^2^, ^10^, ^27^, ^28^ In this work, we report a film electrode based on dual‐confined FeOOH QDs (denoted FQDs) with superior pseudocapacitive behavior in the 1‐ethyl‐3‐methylimidazolium tetrafluoroborate (EMIMBF_4_) IL electrolyte, where pseudocapacitive FQDs (≈5 nm) are steadily dual confined in a 2D heterogeneous nanospace supported by graphite carbon nitride (g‐C_3_N_4_) and Ti‐MXene (Ti_3_C_2_). Probing of the potential‐driven ion accumulation elucidates that strong adsorption occurs between the IL cation (EMIM^+^) and the electrode surface with abundant active sites, providing sufficient redox reaction of FQDs in the film electrode. Furthermore, porous g‐C_3_N_4_ and conductive Ti_3_C_2_ act as ion‐accessible channels and charge‐transfer pathways, respectively, allowing highly dynamic motion of IL ions and electrons. As a result, the dual‐confined FQDs‐based film (denoted FQDs/CNTC film) as a binder‐free electrode delivers a large specific capacitance (≈391.78 F cm^−3^) with high durability in the IL electrolyte. Based on this, a high‐voltage flexible SC (FSC) based on an ionogel electrolyte is fabricated, which exhibits a high energy density (77.12 mWh cm^−3^), a high power density (≈6000 mW cm^−3^), and a remarkable rate and cycling performance. For a real application, the FSC can be charged by harvesting sustainable energy and effectively power various wearable and portable electronics.

## 2 Results and Discussion

**Figure 1:**
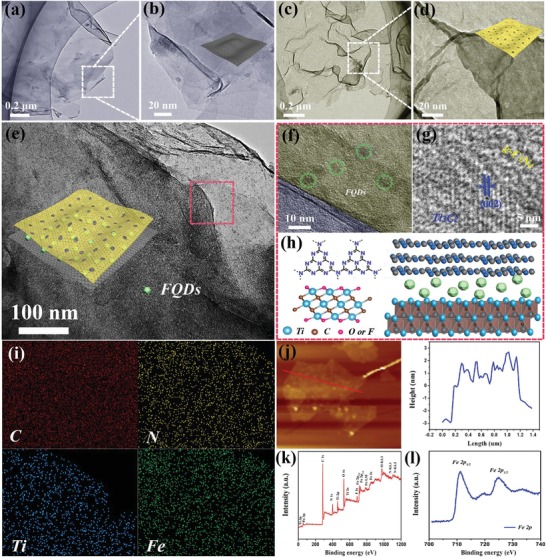
a) Low‐resolution and b) high‐resolution TEM images of conductive Ti_3_C_2_ nanosheets. c) Low‐resolution and d) high‐resolution TEM images of porous g‐C_3_N_4_ nanosheets. e) TEM image of FQDs/CNTC. f,g) High‐resolution TEM images of the red dotted area in (e). h) Structural schematic diagram of FQDs/CNTC dual supported by g‐C_3_N_4_ and Ti_3_C_2_. i) Elemental mapping images and j) AFM image of FQDs/CNTC. k) XPS full spectrum and l) Fe 2p high‐resolution XPS spectrum of FQDs/CNTC.

High‐conductivity Ti_3_C_2_ nanosheets were successfully prepared by etching the Al layers in Ti_3_AlC_2_ using a mixture of LiF and HCl, followed by liquid sonication exfoliation (Figure S1, Supporting Information). As observed in **Figure** **1**a, the Ti_3_C_2_ nanosheets are transparent with lateral dimensions of several micrometres. The magnified image reveals the wrinkled silk‐like texture of the Ti_3_C_2_ nanosheets with an average thickness of 2 nm (Figure 1b; Figure S2, Supporting Information). Moreover, porous g‐C_3_N_4_ nanosheets were synthesized by a two‐step heat treatment involving oxidized etching for pore formation and thermal oxidation for exfoliation (Figure S3, Supporting Information). As shown in Figure 1c, porous g‐C_3_N_4_ is composed of ultrathin crumpled nanosheets (≈3 nm, obtained from Figure S4, Supporting Information), in which abundant porous structures exist in the layers. These pores are abundant and continuous, while the ultrathin pore wall (Figure 1d) provides numerous facile accessible channels and a short diffusion distance for electrolyte ions.^29^, ^30^, ^31^ The X‐ray diffraction (XRD) results of g‐C_3_N_4_ and Ti_3_C_2_ nanosheets are provided in Figures S5 and S6 of the Supporting Information.

Due to the similar planar nanosheets of porous g‐C_3_N_4_ and conductive Ti_3_C_2_, FQDs/CNTC was prepared, yielding FQDs confined in g‐C_3_N_4_ and Ti_3_C_2_ sandwich‐type nanosheets. The TEM image of FQDs/CNTC in Figure 1e reveals that a large amount of FQDs are uniformly dispersed and sandwiched within the interlayer of the nanosheets, in which the size of the FQDs is ≈5 nm (Figure 1f). The high‐resolution TEM image in Figure 1g shows lattice fringes with a spacing of 1 nm, corresponding to the (002) plane of Ti_3_C_2_,^32^ whereas the disordered area is ascribed to g‐C_3_N_4_.^33^ These results strongly indicate that the sandwich‐type nanosheets are constructed by g‐C_3_N_4_ and Ti_3_C_2_, thus offering a heterogeneous nanospace for effective dual confinement of FQDs (Figure 1h). More information about the structural characterizations of FQDs in FQDs/CNTC is provided in Figures S7–S9 of the Supporting Information. The natural structure of g‐C_3_N_4_ contains a large amount of pyrrolic N “hole” defects in the lattice and doubly bonded N at the vacancy edges (Figure S10, Supporting Information),^34^, ^35^ while Ti_3_C_2_ possesses some surface functional groups (Ti–O and Ti–F) due to its etching process with some chemical agents (Figure S11, Supporting Information),^36^, ^37^ both of which are beneficial for the adsorption and movement of electrolyte ions. As shown in Figure 1i, the corresponding elemental mapping analysis of the constituent elements C, N, Ti, and Fe clearly displays a well‐defined compositional profile of FQDs/CNTC, further manifesting the formation of FQDs in the heterogeneous nanospace consisting of g‐C_3_N_4_ and Ti_3_C_2_. This result is also supported by the atomic force microscopy (AFM) image (Figure 1j) of FQDs/CNTC with a height of 5–5.5 nm, which results from g‐C_3_N_4_ (≈3 nm thickness) and Ti_3_C_2_ (≈2 nm thickness). In addition, the X‐ray photoelectron spectroscopy (XPS) full spectrum (Figure 1k) reveals that the FQDs/CNTC contains C, N, Ti, Fe, and O atoms. As shown in the high‐resolution Fe 2p spectrum (Figure 1l), two main peaks are located at binding energies of 711.3 and 724.8 eV for Fe 2p_3/2_ and Fe 2p_1/2_, which are characteristic of the Fe^3+^ of FeOOH in FQDs.^38^ Meanwhile, the FQDs exhibit amorphous feature according to the XRD result (Figure S12, Supporting Information). The electrochemical activity of amorphous‐state FeOOH has been demonstrated to be better than that of crystalline‐state FeOOH with similar particle size and morphology.^39^, ^40^

For flexible SC application, a freestanding and binder‐free FQDs/CNTC film electrode was obtained via facile vacuum‐induced filtration. The elimination of an insulating polymeric binder could increase the utilization of electroactive material and enhance the electrical conductivity of the electrode. As shown in Figure S13 of the Supporting Information, the resultant FQDs/CNTC film exhibits a well‐aligned layered and a compact structure, in which the robust skeleton is dual supported by g‐C_3_N_4_ and Ti_3_C_2_, thus providing the FQDs/CNTC film with high mechanical stability for use as a flexible electrode (Figure S14, Supporting Information). By contrast, FQDs/g‐C_3_N_4_ and FQDs/Ti_3_C_2_ show poor film forming ability, which are both easily disintegrated after vacuum filtration (Figure S15, Supporting Information). Therefore, the freestanding FQDs/CNTC film electrode can be readily applied in flexible energy‐storage devices, catering to the need for portable and wearable electronic technologies.

**Figure 2:**
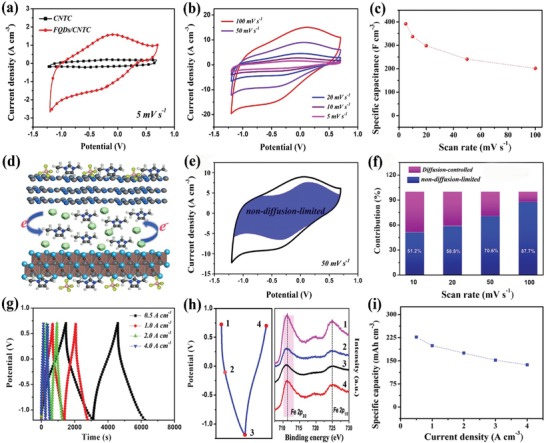
a) Comparison of CV curves scanned at 5 mV s^−1^ of the CNTC and FQDs/CNTC film electrodes in the EMIMBF_4_ IL electrolyte. b) CV curves and c) corresponding specific capacitance of the FQDs/CNTC film electrode at various scan rates (5–100 mV s^−1^). d) Schematic of the energy‐storage behavior of the FQDs/CNTC film electrode in the EMIMBF_4_ IL electrolyte. e) Voltammetric response, with the shaded region representing the non‐diffusion‐limited storage. f) Contributions of diffusion‐controlled and non‐diffusion‐limited storage at different scan rates. g) GCD curves at various current densities of the FQDs/CNTC film electrode in the EMIMBF_4_ IL electrolyte. e) XPS Fe 2p spectra of the FQDs/CNTC film electrode at selected charge/discharge states. i) Corresponding specific capacity under different current densities.

The electrochemical behaviors of the CNTC (only g‐C_3_N_4_ and Ti_3_C_2_) and FQDs/CNTC film electrodes were evaluated using the three‐electrode configuration in the EMIMBF_4_ IL electrolyte. As shown in **Figure** **2**a, the cyclic voltammetry (CV) curve of the FQDs/CNTC film electrode exhibits broad and obvious redox peaks at −0.25 V (cathodic peak) and −0.05 V (anodic peak), showing a higher specific capacitance than the CNTC film electrode with limited capacitive storage (detailed description in Figure S16, Supporting Information) in the IL electrolyte. Figure 2b shows that the set of redox peaks is visible for the anodic and cathodic sweeps in various CV curves of the FQDs/CNTC film electrode, indicating its dominant pseudocapacitive storage in the IL electrolyte. As calculated using Equation (4), the specific capacitance of the FQDs/CNTC film electrode based on the total volume at 5 mV s^−1^ can reach 391.78 F cm^−3^, considerably larger than that of previously reported film electrodes (mostly <250 F cm^−3^) in IL electrolytes.^41^, ^42^, ^43^, ^44^, ^45^ When the scan rate increases to 100 mV s^−1^, the specific capacitance still remains at 201.22 F cm^−3^ (Figure 2c).

With regard to the FQDs/CNTC film electrode, the CNTC with capacitive storage (≈46 F cm^−3^ at 5 mV s^−1^) certainly contributes to the total capacitance, but the total capacitance is mainly contributed by the pseudocapacitive redox reaction of FQDs in the FQDs/CNTC film electrode (Figure 2d). As shown in Figure 2e,f, the non‐diffusion‐limited storage in the FQDs/CNTC film electrode is prominent, indicating that the redox reaction originates from the fast pseudocapacitive process instead of the slow diffusion‐controlled battery‐type energy storage. The nonlinear curves without a plateau (Figure 2g) further indicate that the FQDs/CNTC film electrode mainly stores pseudocapacitive charge in the IL electrolyte, similar to the reported pseudocapacitive electrodes for SCs.^46^, ^47^ To elucidate the evolution of FQDs in the film electrode during the electrochemical process, the chemical state variation of the Fe element was investigated through XPS analysis at selected charge/discharge states (Figure 2h). The main peak of Fe 2p_3/2_ at 711.3 eV slightly shifts to a lower binding energy with deepening of discharge owing to the partial conversion of trivalent Fe to divalent Fe.^48^ In turn, during the charging process, the valence state of the reduced bivalent Fe can change to trivalent Fe, further indicating the reversible redox reaction of FQDs in the electrode with IL ions to generate and store charge. The pseudocapacitive mechanism is associated with the following reaction: Fe^III^OOH + *x*[EMIM]^+^ + *xe* ↔ Fe^III^Fe^II^_1‐_ *_x_*OOH[EMIM]*_x_* ^+^.^38^, ^48^ The charge stored in the FQDs/CNTC film electrode, or the capacity (mAh cm^−3^), was evaluated using galvanostatic charge–discharge (GCD) curves (on the basis of Equation (5)) at current densities from 0.5 A cm^−3^ to 4 A cm^−3^. The specific capacity of the FQDs/CNTC film electrode is calculated to be 227.02 mAh cm^−3^ at 0.5 A cm^−3^ and 137.64 mAh cm^−3^ at 4 A cm^−3^, or a 60.6% capacity retention in the IL electrolyte.

**Figure 3:**
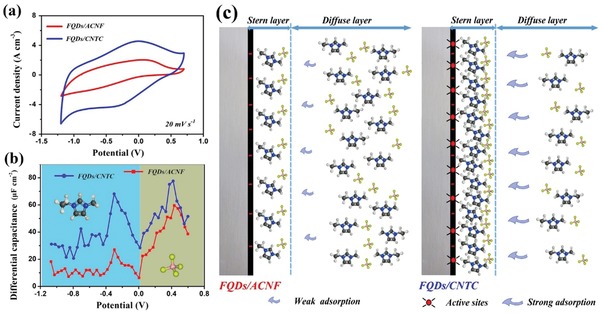
a) CV curves scanned at 20 mV s^−1^, b) *C*_d_–*E* curves, and c) schematic diagrams of stern and diffuse layer structures of FQDs/CNTC and FQDs/ACNF film electrodes in the EMIMBF_4_ IL electrolyte.

To investigate the role of g‐C_3_N_4_ and Ti_3_C_2_ in the FQDs/CNTC electrode, a film electrode of FQDs grown on common high‐temperature activated carbon nanofibers (FQDs/ACNFs) was obtained, and its electrochemical behavior was compared with that of the FQDs/CNTC electrode in the EMIMBF_4_ IL electrolyte. **Figure** **3**a displays the CV curves of the FQDs/CNTC and FQDs/ACNF film electrodes at a scan rate of 20 mV s^−1^. Obvious redox peaks are observed in the anodic and cathodic sweeps, suggesting that the dominant pseudocapacitive storage of the FQDs/CNTC and FQDs/ACNF film electrodes in the IL electrolyte results from the reversible redox reactions of the FQDs in the electrodes with IL ions (EMIM^+^). In addition, the FQDs/CNTC film electrode shows higher peak currents and a larger CV area, thus revealing sufficient redox reaction and superior pseudocapacitive behavior of the FQDs/CNTC film electrode in the IL electrolyte.

Potential‐driven ion accumulation can be probed using the differential capacitance, which has been regarded as an effective indicator of the IL ion density near the electrode, and interpreted based on Kornyshev theory.^49^ Therefore, the differential capacitance of FQDs/CNTC and FQDs/ACNF film electrodes in the EMIMBF_4_ IL electrolyte were investigated using *C*_d_–*E* curves.^49^, ^50^ Electrostatic interactions cause counterions to accumulate on the surface of the charged electrode, forming a compact stern layer and a loose diffuse layer.^51^ In the *C*_d_–*E* curve, the differential capacitance depends on the accumulated ion density in the stern layer.^49^, ^50^, ^52^ Figure 3b shows the *C*_d_–*E* curves of FQDs/CNTC and FQDs/ACNF film electrodes in the EMIMBF_4_ IL electrolyte, both displaying two obvious peaks in the curves. According to Kornyshev theory, for camel‐shaped curves, the potential of zero charge (PZC) is the potential corresponding to the minimum capacitance between the two peaks.^53^, ^54^ When the electrode potential negatively moves away from the PZC, the cations move towards the stern layer to shield the negative charge on the electrode surface (more details are provided in Figure S17, Supporting Information). For the EMIMBF_4_ IL electrolyte (Figure S18, Supporting Information), movement of the larger‐sized EMIM^+^ cations in the diffuse layer and accumulation in the stern layer are more difficult compared with the smaller BF_4_^−^ anions.^53^, ^55^ As a result, the differential capacitance of the FQDs/ACNF electrode is lower under negative polarization than positive polarization, indicating its inefficient EMIM^+^ accumulation in the stern layer. However, EMIM^+^ is the working ion triggering the pseudocapacitive reaction with FQDs in the electrode, so the unsatisfactory pseudocapacitive storage of the FQDs/ACNF electrode is due to the lack of EMIM^+^ for sufficient redox reaction with FQDs in the FQDs/ACNF electrode (the left model in Figure 3c). By contrast, the *C*_d_–*E* curve of the FQDs/CNTC electrode is nearly asymmetric, in which the differential capacitance is significantly increased when the electrode is negatively charged, indicating a high‐accumulated EMIM^+^ ion density in the stern layer. This phenomenon is mainly due to the abundant active sites consisting of numerous lone pair electrons, such as N defects of g‐C_3_N_4_ and surface functionality of Ti_2_C_3_ in the FQDs/CNTC electrode, which easily overlap with the electron cloud of the positively charged imidazole ring. The overlap of electron clouds could effectively form strong adsorption between EMIM^+^ cations and the electrode surface, leading to large numbers of EMIM^+^ cations being adsorbed and arranged in the stern layer over a short distance (the right model in Figure 3c). The higher the EMIM^+^ cation accumulation, the more these cations fully participate in the redox reaction with FQDs, leading to the FQDs/CNTC film electrode exhibiting excellent pseudocapacitive behavior.

**Figure 4:**
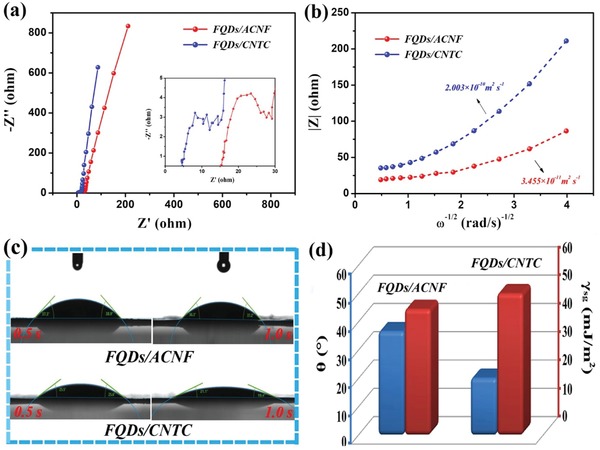
a,b) Comparison of Nyquist plots (a) and diffusion coefficient curves (b) of the FQDs/CNTC and FQDs/ACNF film electrodes in the EMIMBF_4_ IL electrolyte. c) Contact angle of EMIMBF_4_ IL droplets on the FQDs/ACNF and FQDs/CNTC film electrodes. d) Corresponding contact angle and solid surface free energy.

Electrochemical impedance spectroscopy (EIS) is a powerful technique that provides much information regarding the electrochemical kinetic characteristics of the FQDs/CNTC and FQDs/ACNF film electrodes in the EMIMBF_4_ IL electrolyte. The point intersecting the real axis in the high frequency region represents the equivalent series resistance (*R*_s_), which reveals the total inner resistance of the electrode.^56^ As shown in the inset of **Figure** **4**a, the *R*_s_ of the FQDs/CNTC film electrode is lower than that of the FQDs/ACNF film electrode owing to the ultrahigh electrical conductivity of Ti_3_C_2_ in the FQDs/CNTC film electrode. Moreover, the diameter of the semicircle in the high frequency region is related to the charge‐transfer resistance (*R*_ct_), and the straight‐line part in the low‐frequency region represents the Warburg impedance (*Z*_w_),^57^ which is described as the diffusive impendence of the IL ions within the film electrode. Clearly, the FQDs/CNTC film electrode shows a smaller semicircle and a more vertical straight line (Figure 4a), indicating the favorable kinetic behavior of ionic diffusion and charge transport for the FQDs/CNTC film electrode in the IL electrolyte. To deeply analyse the IL ion dynamics, the diffusion coefficient of IL ions in the film electrode can be estimated using impedance data at a low frequency. In this frequency range, the magnitude of the impedance varies linearly with ω^−1/2^, in accordance with Equation (1), 58, 59

(1) $$Z\;=\;\frac{L}{C_{L}{\left(D\cdot \omega \right)}^{1/2}}$$

where *D*, *C*_L_, and *L* are the diffusion coefficient, low‐frequency redox capacitance, and film thickness, respectively. The value of *C*_L_ can be obtained from the plot of −*Z*_im_ versus 1/ω, which is linear with a slope of *1*/*C*_L_, in accordance with Equation (2)

(2) $$-Z_{\mathrm{im}}\;=\;\frac{1}{C_{L}\omega }$$

As shown in Figure S19 of the Supporting Information, the *C*_L_ values of the FQDs/ACNF and FQDs/CNTC film electrodes are calculated to be 19 and 25 mF, respectively. By substituting the latter value into Equation (1), the diffusion coefficient of the IL ions in the FQDs/CNTC film electrode is calculated to be 2.003 × 10^−10^ m^2^ s^−1^ (Figure 4b), which is much higher than that in the FQDs/ACNF film electrode (3.455 × 10^−11^ m^2^ s^−1^), further confirming the enhanced IL ion dynamics of the FQDs/CNTC film electrode.

Wettability is one of the most crucial properties of a film electrode. The microstructure of the film electrode influences its wettability to a large extent. For a liquid droplet on a flat solid, the wetting behavior is determined by Young's equation^60^, ^61^

(3) $$\gamma_{\mathrm{sl}}\;=\;\gamma_{\mathrm{sg}}-\gamma_{\mathrm{lg}}\cdot \cos\theta$$

The contact angle (θ), the surface tension (γ_lg_) of the liquid, the surface free energy (γ_sg_) of the solid, and the interfacial free energy (γ_sl_) between the solid and liquid are related. Figure 4c shows the wetting process of EMIMBF_4_ IL droplets in contact with the FQDs/ACNF and FQDs/CNTC film electrodes. The FQDs/CNTC film electrode is almost completely wetted by the IL electrolyte, exhibiting significantly better wettability than the FQDs/ACNF film electrode. The small θ (20.3°) and large γ_sg_ (50.2 mJ m^−2^) of the FQDs/CNTC film electrode in the IL electrolyte (Figure 3d) both confirm its excellent wettability. The γ_lg_ of the EMIMBF_4_ IL electrolyte is measured to be ≈52.4 mJ m^−2^, and the γ_sl_ of the FQDs/CNTC film electrode in the IL electrolyte is calculated to be 1.05 mJ m^−2^, which is ≈2.6 times lower than that of the FQDs/ACNF film electrode (2.71 mJ m^−2^). The low γ_sl_ value indicates strong adhesion between the FQDs/CNTC film electrode and the IL electrolyte, which is mainly due to the abundant pore channels (Figure S20, Supporting Information) and active sites of the FQDs/CNTC film electrode. As shown in Table S1 of the Supporting Information, the calculated liquid electrode uptake (EU) and porosity (P) of the FQDs/CNTC film are higher than those of the FQDs/ACNF film, further indicating the superior wettability of the FQDs/CNTC film in the EMIMBF_4_ IL electrolyte. Furthermore, the cycling stability of the FQDs/CNTC film electrode with a high retention of 85.2% after 10 000 cycles is shown in Figure S21 of the Supporting Information. More information about electrochemical performance comparisons of FQDs/g‐C_3_N_4_, FQDs/Ti_3_C_2_ (common coating method) and FQDs/CNTC film electrodes in the IL electrolyte is provided in Figure S22 of the Supporting Information.

**Figure 5:**
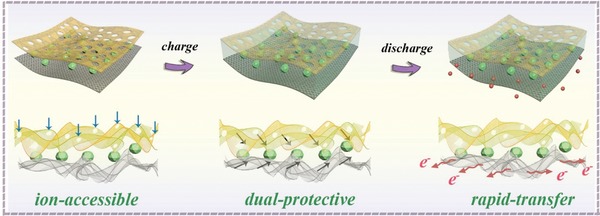
Schematic of the electrochemical process of the FQDs/CNTC film electrode in the IL electrolyte.

The superior electrochemical energy storage of the FQDs/CNTC film electrode in the IL electrolyte is schematically illustrated in **Figure** **5**, which can be attributed to the following aspects. (1) Abundant active sites consisting of numerous lone pair electrons, such as nitrogen defects of g‐C_3_N_4_ and surface functionality of Ti_2_C_3_, are favorable for the adsorption of IL ions. Meanwhile, the rich porous structure of g‐C_3_N_4_ provides accessible channels for IL ions (blue arrows). Both these factors ensure that IL ions are involved in the sufficient redox reaction with FQDs, leading to the FQDs/CNTC film electrode exhibiting a high specific capacitance. (2) FQDs are dual confined in g‐C_3_N_4_ and Ti_3_C_2_ heterogeneous nanosheets, forming a robust sandwich‐type architecture. This can provide a suitable nanospace for the dual protection of FQDs (black arrows), which effectively inhibits the aggregation and dissolution of FQDs during the cycling process, rendering the high cycle stability of the FQDs/CNTC film electrode. (3) Highly conductive Ti_3_C_2_ acts as a “highway” to facilitate efficient charge transport (red arrows), which benefits the rate capability of the FQDs/CNTC film electrode in the IL electrolyte.

**Figure 6:**
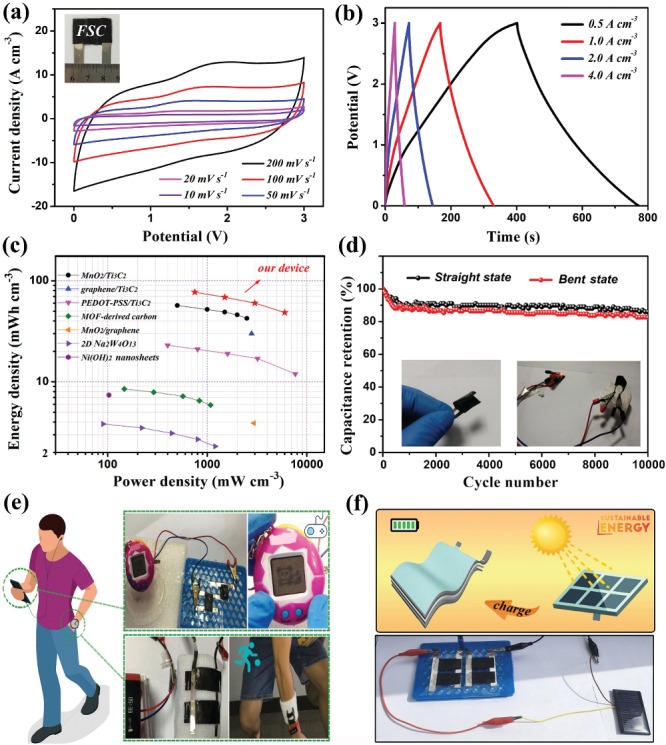
a) CV curves of the FSC at various scan rates. The inset is a digital photo of the FSC. b) GCD curves and c) Ragone plot of the FSC. d) Cycle life of the FSC in the straight and bent states at a current density of 4.0 A cm^−3^ after 10 000 cycles. The inset shows the power supply of the FSC under the bent state. e) Digital photos showing the power supply of two FSCs in series or parallel connection for various portable and wearable electronics. f) Schematic diagram and real‐time image of four FSCs charged by harvesting sustainable solar energy in real life.

Similar to the IL electrolyte, an ionogel as a solid‐state electrolyte exhibits nonvolatility, nonflammability, high thermal stability, and a wide electrochemical potential window (Figure S23, Supporting Information).^16^, ^62^ Hence, a flexible SC (FSC) has been fabricated with an ionogel electrolyte using poly(vinylidene fluoride‐hexafluoropropylene) (P(VDF‐HFP)) copolymer as a polymer matrix and hydrophobic EMIMBF_4_ IL as a solvent. Differentiated by the stable potential ranges in the EMIMBF_4_ IL electrolyte (Figure S25, Supporting Information), the FQDs/CNTC film and CNTs/RGO film (Figure S24, Supporting Information) are used as negative and positive electrodes, respectively, in the device. As seen from the CV curves in **Figure** **6**a, an FSC with a wide potential window of 3.0 V and a large specific capacitance of 71.26 F cm^−3^ (calculated by Equation (6)) is achieved at 10 mV s^−1^. Figure 6b shows the GCD curves of the FSC in the range of 0.5–4 A cm^−3^. As calculated using Equations (7) and (8), the maximum energy density based on the total volume of the FSC (≈0.0105 cm^3^, configuration illustrated in Figure S26, Supporting Information) is 77.12 mWh cm^−3^ at a power density of 750 mW cm^−3^. Additionally, the maximum power density of the FSC is ≈6000 mW cm^−3^ at an energy density of 48.49 mWh cm^−3^, which is much better than those of previously reported state‐of‐the‐art FSCs (Figure 6c).^63^, ^64^, ^65^, ^66^, ^67^, ^68^, ^69^ In addition, the cycling stabilities of the fabricated FSC in the straight and bent states reveal a high retention above 80% after 10 000 cycles (Figure 6d). Importantly, the FSC is highly flexible, mechanically stable, and randomly bendable without destroying the structural integrity or electrochemical performance of the device (Figures S27 and S28, Supporting Information). During the deformation, the device can still light up a lamp (1 W, 2.0 V) with a bright red color (inset in Figure 6d).

To further demonstrate practical potential applications, we integrated the FSC as an energy‐storage device to power some common electronic products in real life. As is notably observed in Figure 6e, two FSCs are connected in a series or parallel arrangement, which can easily power portable and wearable electronics, such as handheld game players and smart bracelets. More importantly, the FSCs as efficient energy‐storage devices can power electronic products in complex motion states. This result indicates the great electrochemical reliability of the FSCs with high flexibility. Apparently, the FSCs are practically promising as a supplemental power source replacing microbatteries. Furthermore, an attempt to store renewable solar energy in FSCs was made, in which sustainable energy could be harvested for portable and wearable electronics. A commercial miniature solar‐cell panel (5.5 V, 20 mA) was assembled using the fabricated FSCs as a sustainable energy system. Four FSCs in a series and parallel arrangement serve as an energy‐storage module (Figure 6f), collecting solar energy generated by the solar‐cell panel. After charging, the FSCs with high energy density could effectively power electronic products. The corresponding videos are shown in Movie S1 of the Supporting Information. The self‐discharging performance is a crucial parameter for the practical application of FSCs. As shown in Figure S29 of the Supporting Information, the fabricated FSCs present a rapid self‐discharge process in the first few minutes that gradually slows down over several hours. Finally, the open‐circuit voltage of the devices is maintained at ≈2.5 V beyond 10 h, indicating a low self‐discharge rate of the fabricated FSCs after harvesting sustainable energy. Therefore, this study introduces a new avenue for flexible energy‐storage devices, which is of great significance for the development of sustainable wearable and portable electronics.

## 3 Conclusions

We have successfully developed a new strategy to obtain a unique FQDs/CNTC film electrode, which was realized by the dual confinement of pseudocapacitive FQDs in porous g‐C_3_N_4_ and conductive Ti_3_C_2_ nanosheets. Porous g‐C_3_N_4_ and conductive Ti_3_C_2_ nanosheets not only act as ion‐accessible channels and charge‐transfer pathways, respectively, but also jointly offer a heterogeneous nanospace for the dual protection of FQDs. Probing of the potential‐driven ion accumulation elucidated that strong adsorption can occur between the IL cation (EMIM^+^) and the electrode surface with abundant active sites, providing sufficient pseudocapacitive behavior of the FQDs/CNTC film electrode in the IL electrolyte. As a consequence, a 3 V high‐voltage FSC could be fabricated using an ionogel as a solid‐state electrolyte, which presented a high energy density (77.12 mWh cm^−3^), a high power density (6000 mW cm^−3^), and a remarkable rate and cycling performance. The FSCs could effectively power various wearable and portable electronics, making them suitable for a wide range of applications in stylish energy‐storage devices. Considering the facile fabrication and outstanding performance, this work provides new insight into fabricating superior pseudocapacitive QDs electrode materials for flexible energy‐storage devices towards portable and wearable electronics.

## 4 Experimental Section

*Synthesis of Porous g‐C_3_N_4_*: Briefly, 5 g melamine was put into a crucible and heated at 520 °C for 4 h in air to obtain an agglomerate (bulk g‐C_3_N_4_). Then, 1 g bulk g‐C_3_N_4_ powder was placed in an open crucible, followed by heat treatment at 550 °C for 3 h in air with a heating rate of 10 °C min^−1^. Afterward, the obtained powder was dispersed in isopropanol, sonicated for 0.5 h and then separated by centrifugation at ≈5000 rpm to remove the residual incompletely exfoliated part, finally obtaining yellowish‐white porous g‐C_3_N_4_ under vacuum drying.

*Synthesis of Ti_3_C_2_*: Typically, 1 g Ti_3_AlC_2_ was slowly added to a mixed aqueous solution of LiF (1.82 g) and 20 mL HCl (10 m). An ice water bath was used to avoid overheating due to the exothermic reaction. Subsequently, the solution was kept at 38 °C for 48 h to etch the Al in Ti_3_AlC_2_. Afterward, the resultant Ti_3_C_2_ was collected via centrifugation, washed, and dispersed in deionized water to form a homogeneous solution by liquid‐phase ultrasonication.

*Preparation of FQDs/CNTC Film*: First, 40 mg porous g‐C_3_N_4_ was added to 50 mL ethanol with sonication for 5 min. Then, 1 mmol FeCl_3_·6H_2_O and 3 mmol NH_4_HCO_3_ were dissolved separately in the above solution with stirring for 10 h. Next, 30 mL Ti_3_C_2_ dispersion (2 mg mL^−1^) was added with continuous stirring for another 12 h to obtain FQDs/CNTC, which was then dispersed into 100 mL deionized water to form a homogeneous solution (1 mg mL^−1^) under ultrasonic treatment. Finally, 30 mL FQDs/CNTC solution was vacuum filtered through a cellulose ester filter membrane (pore size of 0.22 µm) and then peeled from the membrane to obtain the FQDs/CNTC film. For comparison, high‐temperature ACNFs film was immersed in 50 mL ethanol containing 1 mmol FeCl_3_·6H_2_O and 3 mmol NH_4_HCO_3_, followed by continuous stirring for 10 h, to obtain FQDs/ACNF film. In addition, CNTC film was prepared using a similar method without the addition of FeCl_3_·6H_2_O and NH_4_HCO_3_. FQDs/g‐C_3_N_4_ and FQDs/Ti_3_C_2_ were also prepared through a procedure similar to that mentioned above. (More experimental details are presented in Figure S22, Supporting Information.)

*Characterization*: Field emission scanning electron microscopy (FEI Sirion 200) and TEM (JEM‐2100F) were carried out to characterize the morphology of the samples. AFM images were recorded on a MultiMode 8 (Bruker) in ScanAsyst mode. XRD patterns were characterized on a powder XRD system with Cu Kα radiation, and XPS measurements were performed on a Kratos AXIS Ultra DLD spectrometer with an Al Kα X‐ray source. Nitrogen absorption and desorption measurements were performed with an Autosorb IQ instrument at 77 K. The specific surface area was calculated using the Brunauer–Emmett–Teller method, and the pore size distribution was determined from the adsorption branch of the isotherm according to density functional theory. The surface tension of the EMIMBF_4_ IL was tested using a surface tensiometer (K100, Germany). The wettability of the films in the IL was measured using a contact angle measuring instrument (DCA300, Germany), in which the surface free energy of the films was determined using the conventional two‐liquid‐phase method.

*Electrochemical Measurements*: All electrochemical tests were performed using a VMP3 multifunctional electrochemical analysis instrument (Bio‐Logic, France) via CV, GCD, and EIS methods. The CV and GCD tests were performed at various scan rates and current densities. The EIS measurements were performed in the frequency range of 0.01 Hz to 100 kHz. Additionally, *C*_d_–*E* curves were obtained from EIS measurements according to the previously reported method based on Kornyshev theory.^49^, ^52^ For flexible SC application, FQDs/CNTC film and CNTs/RGO film (additional details are presented in Figure S19, Supporting Information) were used as negative and positive electrodes, respectively. Then, an ionogel electrolyte was slowly sandwiched between the two film electrodes, which were pressed together for 30 min. The ionogel electrolyte was prepared as follows. First, 0.6 g P(VDF‐HFP) was dissolved in an acetone solution. Then, 2.0 g EMIMBF_4_ IL was added to the resulting solution under stirring until the solution became clear. The resulting viscous solution was cast onto a glass petri dish to evaporate the acetone for 1 h to obtain the ionogel electrolyte.

*Calculations for the Electrochemical Tests*: The specific capacitance of a single electrode (*C*_sp_) was calculated from the anodic scan of the CV curve on the basis of Equation (4)

(4) $$C_{\mathrm{sp}}(F\;\;{\mathrm{cm}}^{-3})\;=\;\frac{\int i\cdot dt}{v\cdot U}$$

Here, *i* is the current change over time *t*, *v* is the volume of a single electrode, and *U* is the voltage window of the CV scan.

According to Gogotsi's previous reports,^46^, ^47^ when the GCD curve is nonlinear, the specific capacity of a single electrode, instead of the capacitance, is evaluated using Equation (5)

(5) $$C(\mathrm{mAh}\;\;{\mathrm{cm}}^{-3})\;=\;\frac{I\;\cdot \Delta t}{3.6}$$

where Δ*t* (s) is the discharging time and *I* (A cm^−3^) is the constant current density.

For the two‐electrode system, the specific capacitance of the fabricated FSC was evaluated on the basis of the CV curve using Equation (6)

(6) $$C_{\mathrm{FSC}}(F\;\;{\mathrm{cm}}^{-3})\;=\;\frac{\int i\cdot \;dt}{v_{\mathrm{total}}\cdot U_{\mathrm{FSC}}}$$

where *v*_total_ is the total volume of film electrodes and *U*_FSC_ is the voltage window of the fabricated FSC.

The energy density of the FSC was calculated on the basis of the discharge branch of the GCD curve using Equation (7), and the power density was further evaluated using Equation (8)

(7) $$E\;\left(\mathrm{mWh}\;\;{\mathrm{cm}}^{-3}\right)\;=\;\frac{1}{3.6}\cdot I_{C}\left(\int_{U_{0}}^{U_{\max}}t\;dU\right)$$

(8) $$P\;\left(\mathrm{mW}\;{\mathrm{cm}}^{-3}\right)\;=\;3600\cdot E/\Delta t$$

where *E* (mWh cm^−3^) is the energy density, *P* (mW cm^−3^) is the power density, *I*_C_ (A cm^−3^) is the constant discharge current density, Δ*t* (s) is the discharge time, and *U*_0_ to *U*_max_ is the voltage window of the FSC.

## Conflict of Interest

The authors declare no conflict of interest.

## Supporting information

Supporting Information

Supplemental Movie 1
