# Supplementary material for: Porous g‐C3N4 and MXene Dual‐Confined FeOOH Quantum Dots for Superior Energy Storage in an Ionic Liquid
Source: Adv Sci (Weinh). 2019 Nov 27;7(2):1901975. doi: 10.1002/advs.201901975 (PMC6974951; doi:10.1002/advs.201901975)
Supplement: Supplementary file 1 — Supporting Information [file ADVS-7-1901975-s001.pdf]

Copyright WILEY-VCH Verlag GmbH & Co. KGaA, 69469 Weinheim, Germany, 2019.

ADVANCED SCIENCE
Open Access

Supporting Information

for *Adv. Sci.,* DOI: 10.1002/advs.201901975

Porous g-C_3N_4 and MXene Dual-Confined FeOOH Quantum Dots for Superior Energy Storage in an Ionic Liquid

Minjie Shi, Peng Xiao, Junwei Lang, Chao Yan, and Xingbin Yan**

Copyright WILEY-VCH Verlag GmbH & Co. KGaA, 69469 Weinheim, Germany, 2019.

Supporting Information

Porous g-C_3N_4 and MXene Dual-Confined FeOOH Quantum Dots for Superior Energy Storage in an Ionic Liquid

Minjie Shi, Peng Xiao, Junwei Lang, Chao Yan and Xingbin Yan**

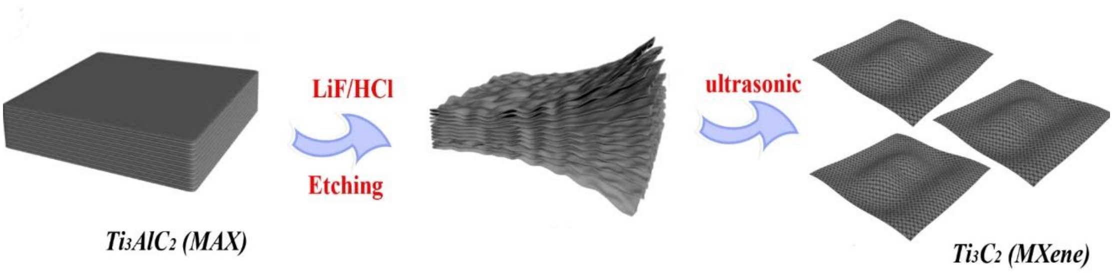

Figure S1. Schematic preparation process of conductive Ti_3C_2 nanosheets.

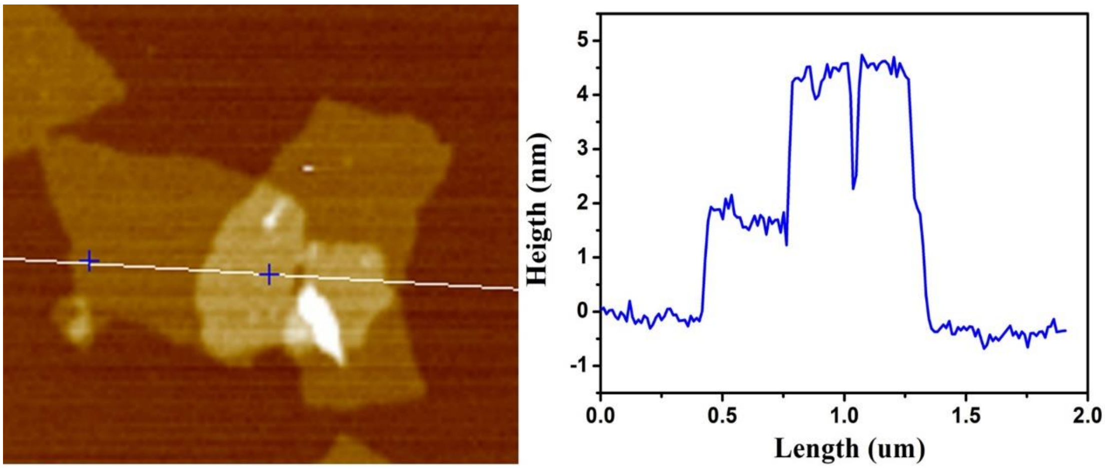

Figure S2. AFM image of Ti_3C_2 nanosheets.

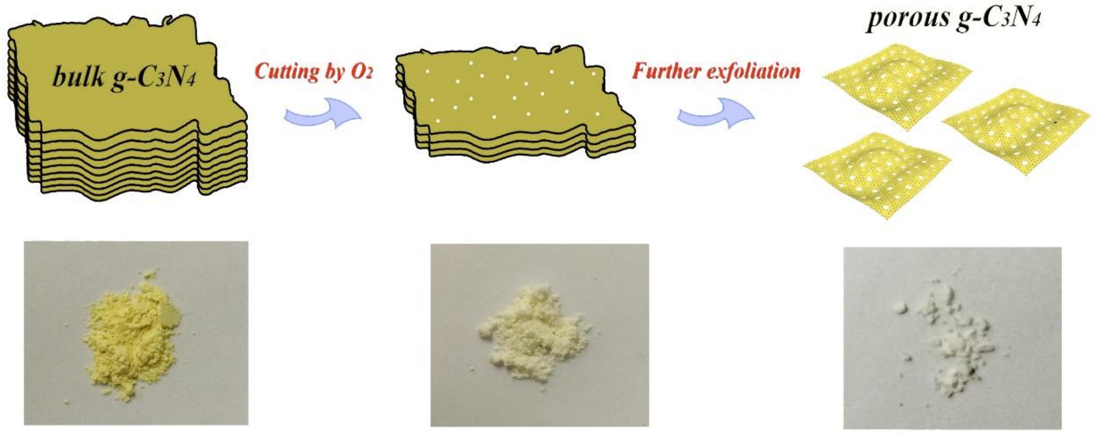

Figure S3. Schematic illustration of the thermal exfoliation to form porous g-C_3N_4 nanosheets.

Porous g-C_3N_4 nanosheets have been synthesized by a two-step thermal treatment which involves oxidized etching for pore digging and thermal oxidation for exfoliation. With the structural defects constituted by heptazine (tris-s-triazine) units in bulk g-C_3N_4, large numbers of pores could be easily formed in the oxidized etching process at high temperature.[1, 2] Besides, hydrogen bonding between strands of polymeric melon units in bulk g-C_3N_4 is not stable enough against thermal oxidation process, thus it could be gradually oxidized from the bulk g-C_3N_4, resulting in ultrathin thickness of g-C_3N_4 nanosheets.[2, 3] The formation of porous g-C_3N_4 nanosheets is hypothesized through a layer-by-layer oxidation cutting for which the schematic illustration is shown in Figure S3. This cutting process through controllable thermal oxidation is hinted by a gradient change of the sample color from yellow to light yellow and lastly to white, which is similar with that of previously reported papers.[4, 5] The reason why the g-C_3N_4 nanosheets appears white is closely related to the increased bandgap from bulk g-C_3N_4 to few-layer g-C_3N_4 nanosheets, which can be ascribed to the strong quantum confinement effect caused by the nature of ultrathin nanosheets.[6, 7]

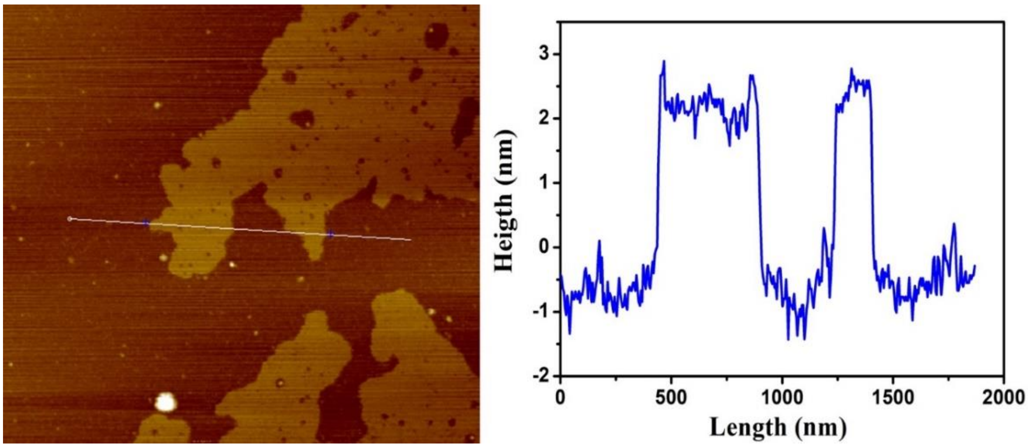

Figure S4. AFM image of porous g-C_3N_4 nanosheets.

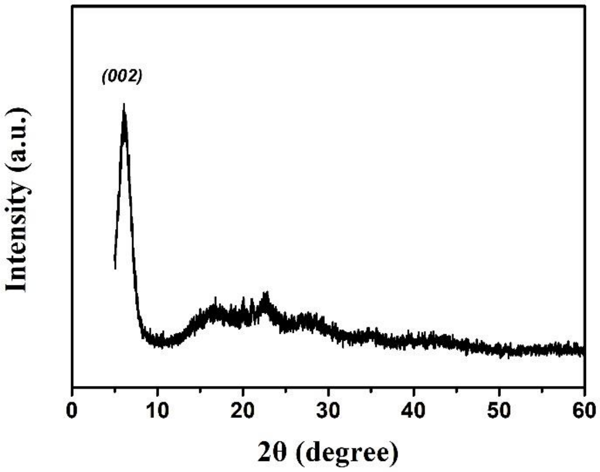

Figure S5. XRD pattern of Ti_3C_2 nanosheets.

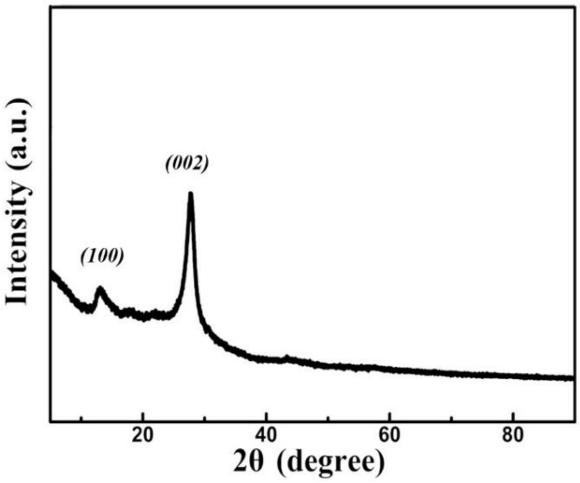

Figure S6. XRD pattern of g-C_3N_4 nanosheets.

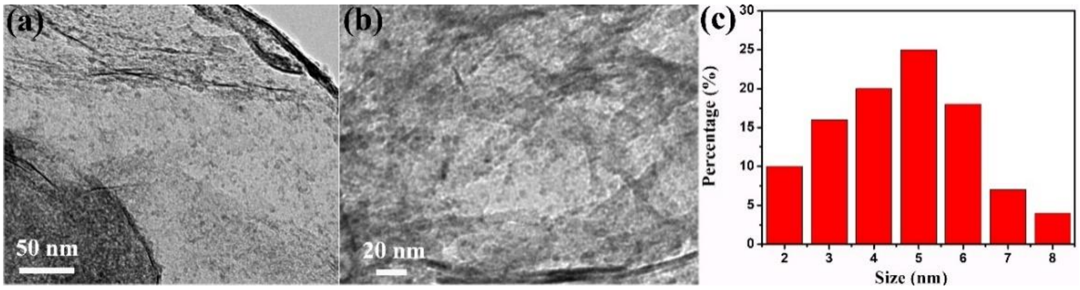

Figure S7. (a) and (b) TEM images with different magnifications, (c) the corresponding size distribution of FQDs in the FQDs/CNTC.

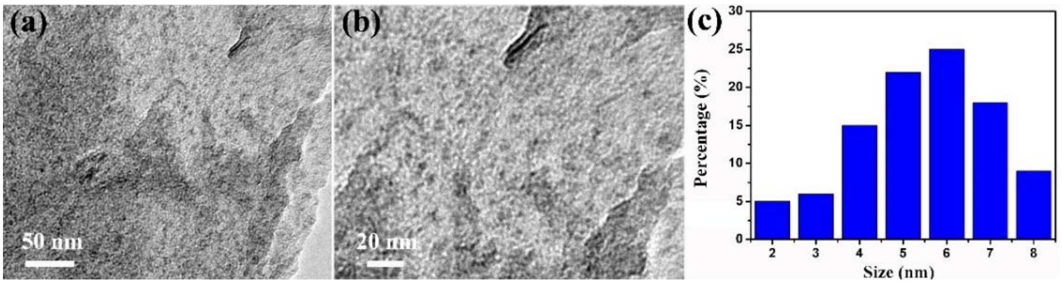

Figure S8. (a) and (b) TEM images with different magnifications, (c) the corresponding size distribution of FQDs in the FQDs/CNTC after cycling test.

TEM images (Figure S7 (a) and (b)) and the corresponding size distribution (Figure S7 (c)) show fairly uniform FQDs with an average size of 5 nm in the FQDs/CNTC. Moreover, TEM measurement (Figure S8 (a) and (b)) of FQDs/CNTC as electrode in IL electrolyte after repeated charging-discharging cycles were also carried out. Clearly, FQDs are uniformly anchor on the constructed nanospace without obvious aggregations in FQDs/CNTC after cycling. Meanwhile, the size distribution (Figure S8 (c)) of FQDs after cycling is slightly broadened with increased percentage of particles with slightly bigger sizes (5~7 nm), indicating the good stability of FQDs confined in nanospace supported by g-C_3N_4 and Ti_3C_2 nanosheets.

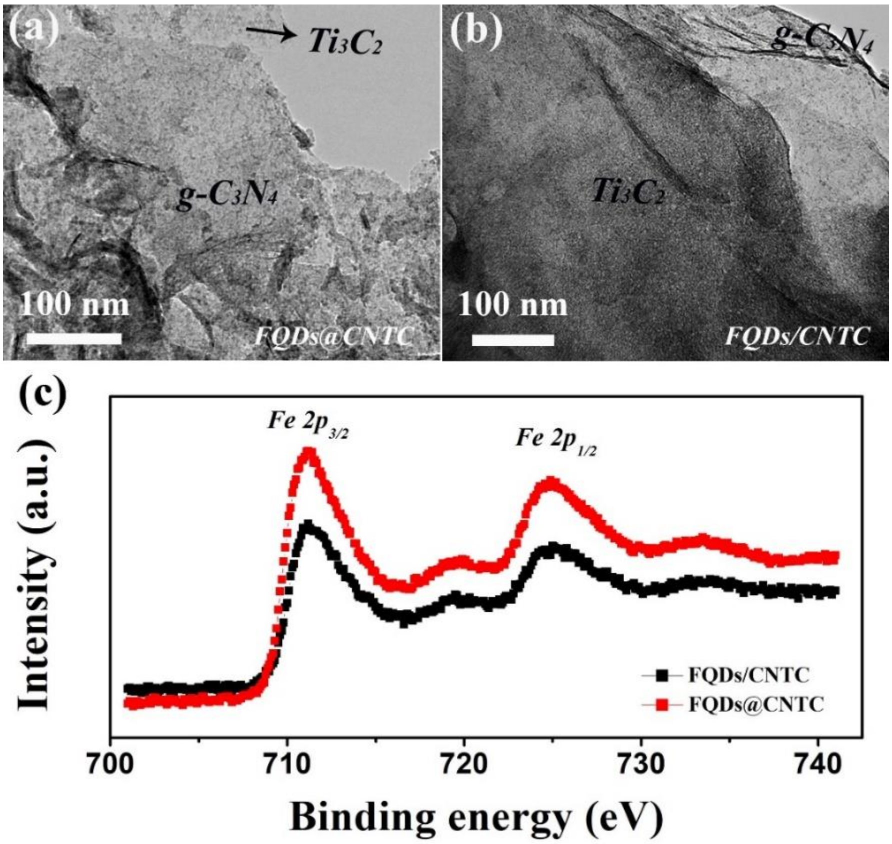

Figure S9. TEM images of (a) FQDs@CNTC and (b) FQDs/CNTC. (c) Fe 2p high-resolution XPS spectra of FQDs/CNTC and FQDs@CNTC.

In our experiment, FeOOH QDs (FQDs) were firstly grown on the porous g-C_3N_4 nanosheets, which were incorporated with Ti_3C_2 nanosheets to form FQDs/CNTC with sandwich-type structure through liquid-phase self-assembly. For comparison, FQDs were directly grown on porous g-C_3N_4 and Ti_3C_2 hybrid nanosheets to obtain FQDs@CNTC. As shown in the TEM image of FQDs@CNTC (Figure S9 (a)), a large number of FQDs are anchored on the surface of porous g-C_3N_4 and Ti_3C_2 nanosheets, which is much different from that of FQDs/CNTC (Figure S9 (b)). Based on the TEM measurement, it is assumed that FQDs could be mainly dispersed between two layers of porous g-C_3N_4 and Ti_3C_2 nanosheets in FQDs/CNTC. In order to further prove this point, high-resolution XPS spectra of Fe 2p were carried out for FQDs/CNTC and FQDs@CNTC. As seen from Figure S9 (c), there are two main peaks located at binding energies of 711.3 and 724.8 eV for Fe $2p_{3/2}$ and Fe $2p_{1/2}$, which are the typical Fe^{3+} characteristics of FQDs in both FQDs/CNTC and FQDs@CNTC. However, the peak intensity of FQDs@CNTC is stronger than that of

FQDs/CNTC, indicating the slight shielding of X-ray photoelectron in Fe 2p spectrum of FQDs/CNTC. This phenomenon might be owing to the FQDs lying between porous g-C_3N_4 and Ti_3C_2 nanosheets in FQDs/CNTC, instead of growing on the surface of nanosheets just like the state of FQDs in FQDs@CNTC, which could lead to the signal attenuation of X-ray photoelectron in Fe 2p spectrum. As a result, FQDs are more inclined to lie between two layers of g-C_3N_4 and Ti_3C_2 nanosheets in FQDs/CNTC, in which g-C_3N_4 and Ti_3C_2 offer a heterogeneous nanospace for effectively dual-confining FQDs, thereby greatly ensuring the superior electrochemical behaviors of FQDs/CNTC as electrode in IL electrolyte.

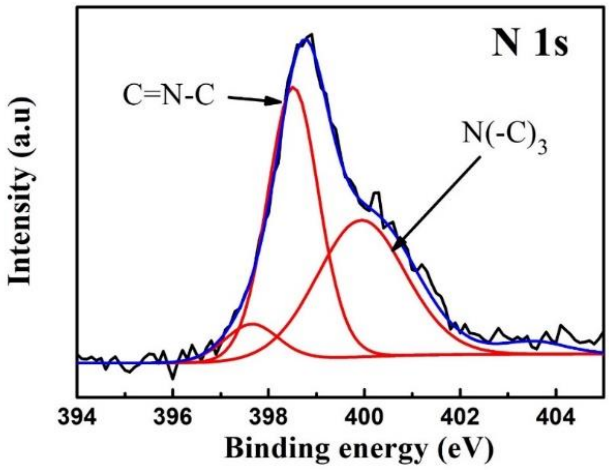

Figure S10. N 1S high-resolution XPS spectrum of FQDs/CNTC.

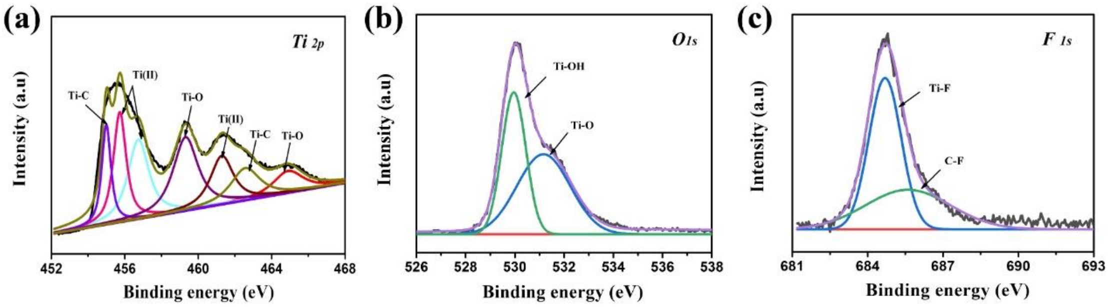

Figure S11 Ti 2p , O 1s and F 1s high-resolution XPS spectrum of FQDs/CNTC.

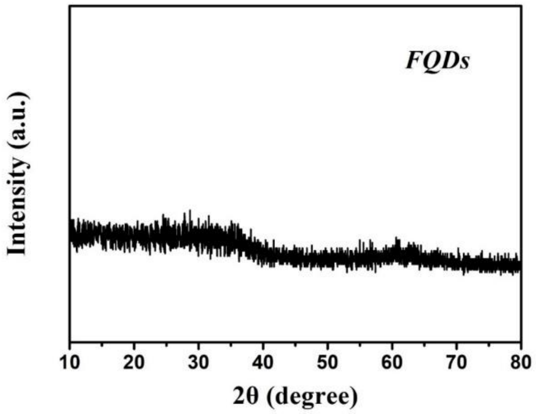

Figure S12. XRD pattern of FQDs. There is no obvious peaks in XRD pattern, indicating the amorphous feature of FQDs.

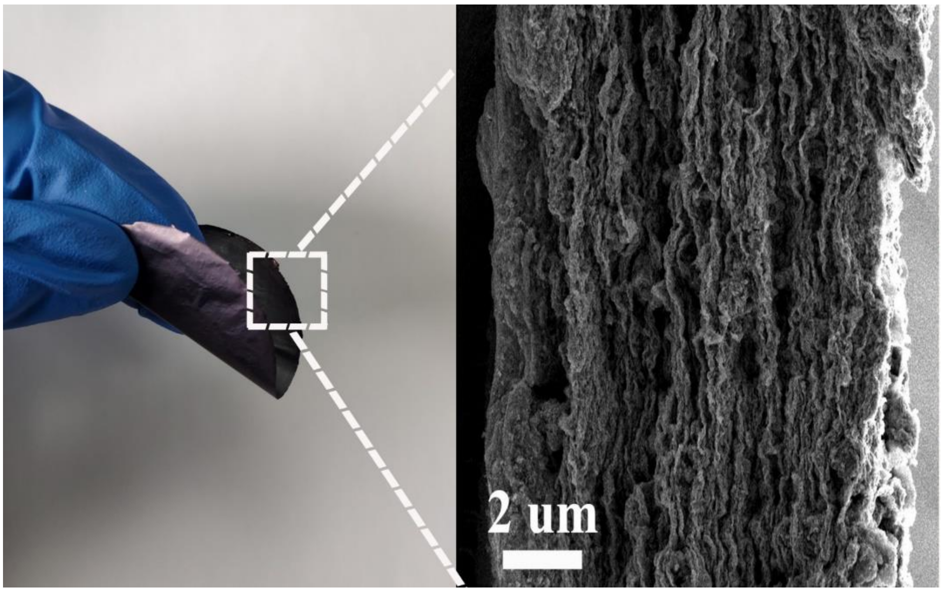

Figure S13. Cross-section FESEM image of FQDs/CNTC film.

Cross-sectional FESEM image reveals the average thickness of FQDs/CNTC film is about 8 μm. The resultant film exhibits a well-aligned layered and compact structure. This dense layered structure ensures the excellent flexibility and mechanical properties of FQDs/CNTC film.

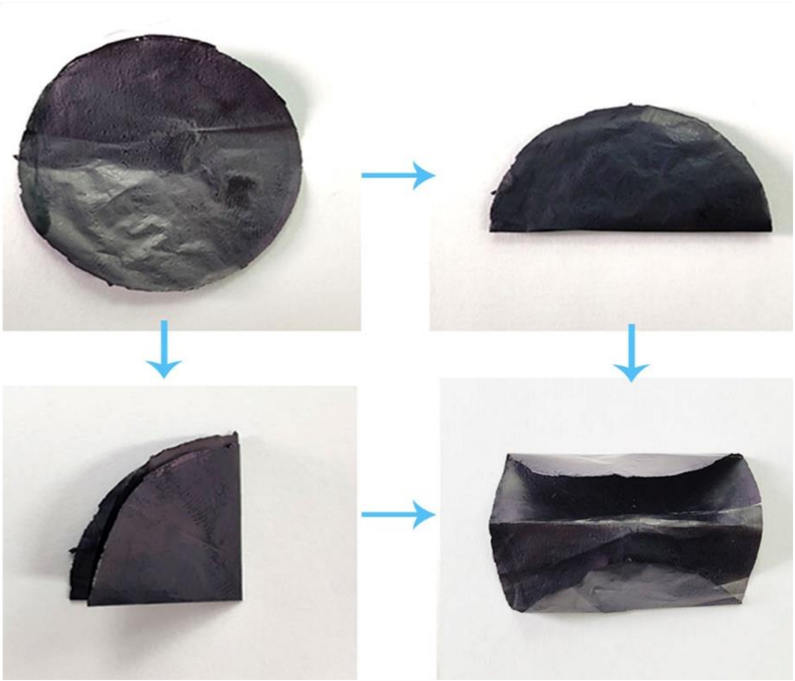

Figure S14. Digital photos of FQDs/CNTC film with high flexibility and editability.

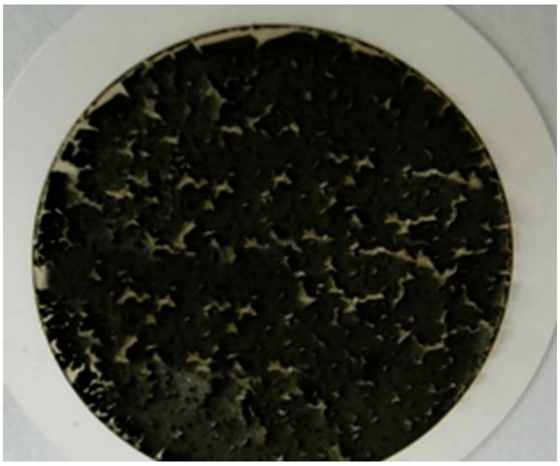

Figure S15. Digital photo of FQDs/g-C_3N_4 and FQDs/Ti_3C_2 with poor film forming ability, which are easily disintegrated after vacuum filtration, so they could not serve as flexible SCs electrodes.

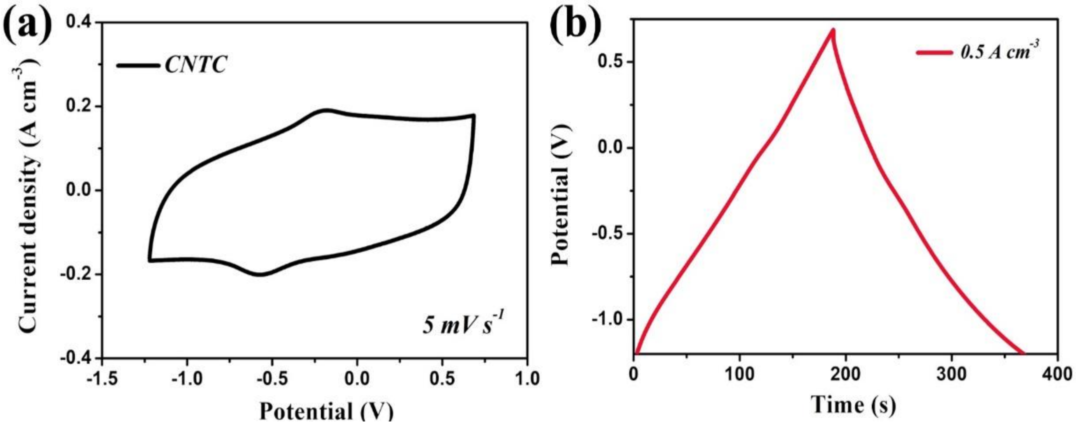

Figure S16. (a) CV curve scanning at 5 mV s^{-1} and (b) GCD curve at a current density of 0.5 A cm^{-3} of CNTC film electrode in $EMIMBF_4$ IL electrolyte.

As shown in Figure S16 (a), the CV curve of CNTC film electrode shows a rectangle-like shape with two small peaks when cycling at 5 mV s^{-1}. The capacitive storage is dominantly occurred in the CNTC film electrode, which is due to that the IL ions electrostatically adsorb on the surfaces of g-C_3N_4 and Ti_3C_2 to form double-layer capacitance, while some pseudocapacitance is resulting from surface redox reaction of functional groups in Ti_3C_2, which are similar with the electrochemical results of surface-functionalized carbon nanomaterial based electrodes. Besides, the nearly linear GCD curve (Figure S16 (b)) also indicates the capacitive storage of CNTC film electrode in IL electrolyte, which is accorded with the characteristics of capacitive-like materials.

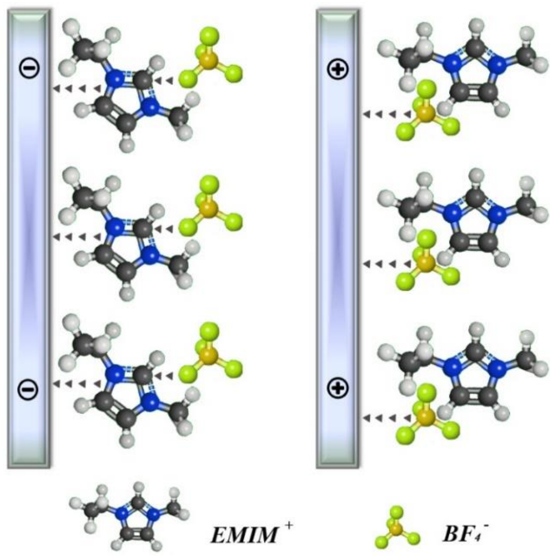

Figure S17. Detailed arrangement in stern layer of $EMIM^+$ cations and BF_4^- anions on

the surface of FQDs/CNTC film electrode.

According to the EIS dates at different potentials, the differential capacitance was estimated by the Equation (1) and the plot of differential capacitance versus electrode potential can be obtained.[8-10]

$$C_d = \frac{1}{j\omega|Z|} \qquad (1)$$

where C_d is the differential capacitance, ω is the angular frequency, $|Z|$ is the lumped impedance and $j^2=-1$. On the basis of Kornyshev theory, the camel-shaped C-E curves with characteristic of two peaks indicates that the $EMIMBF_4$ IL exhibits a small degree of dissociation (γ, defined by the Equation (2)) with a value less than 1/3, which is qualitatively consistent with the dissociation degree of ILs ranging from 0.3 to 0.7 in previously reported literatures.[10, 11]

$$\gamma = \frac{\overline{N}}{N} = \frac{2C_0}{C_{max}} \qquad (2)$$

in which N is the total number of possible configuration of anions and cations, $\overline{N}$ is the total number of anions and cations in the bulk phase, C_0 is the actual concentration of cations or anions, C_{max} is the total concentration of anions and cations accommodated in a certain local range, γ is the degree of dissociation.

As for camel-shaped C-E curves, potential of zero charge (PZC) is the potential corresponding to the minimum capacitance between two peaks. Near PZC, the residual charge on the electrode surface is less, and the neutral alkyl side chain points to the electrode surface. When the electrode potential negatively moves away from PZC, more $EMIM^+$ cations move towards the surface of the electrode to shield the negative charge on the electrode surface. Meanwhile, the $EMIM^+$ cations close to the electrode surface rotate, while the positive charged groups move towards the surface of electrode and the neutral alkyl side chain is far away from the surface of electrode. When the electrode potential continues to move negatively, the alkyl side chains on the electrode surface are completely replaced and the positively charged groups are tightly arranged on the electrode surface, tending to be saturated in the lattice and reaching the maximum differential capacitance. Since the effect of alkyl side chain is equivalent to expanding the distance between the cations and electrode surface, the differential capacitance decreases when the electrode potential continues to move negatively. For positive potential region, the BF_4^- anions replace the neutral alkyl side chains on the electrode surface and the differential capacitance increases with the

increase of the electrode potential. When the electrode potential increases to a certain extent, the alkyl side chains are completely replaced, while the anions are tightly arranged on the surface of the electrode, leading to the maximum differential capacitance.

Electrostatic interaction causes counterions to accumulate to the surface of the charged electrode, which forms a compact Stern layer and a loose diffuse layer. For ILs with small Van der Waals volume, it is easy for the IL ions to move in the diffuse layer and accumulate in the Stern layer, which is favorable for forming more compact Stern layer, thereby resulting in superior interfacial electrochemical behaviors and sufficient pseudocapacitive reaction. This is also demonstrated by previously reported literature about the interfacial differential capacitance investigation of IL electrolyte.[12-16] As a relatively cheap and readily available IL electrolyte, $EMIMBF_4$ IL has been widely used as a suitable electrolyte in energy storage devices, especially SCs applications. Compared with most of IL electrolytes, $EMIMBF_4$ IL exhibits relatively small Van der Waals volume owing to the small size of its cation ($EMIM^+$ ~ 0.75 nm) and anion (BF_4^- ~ 0.51 nm), thus leading to the favorable diffusion of $EMIMBF_4$ IL ions to form Stern layer during the electrochemical reaction. Besides, as shown in Figure S18, $EMIMBF_4$ IL shows extremely high thermal stability (> 350 °C) and wide electrochemical potential window (~ 4 V), while its conductivity can reach as high as 13.4 mS cm^{-1}, which is much higher than most of common ILs (< 10 mS cm^{-1}).[17-19]

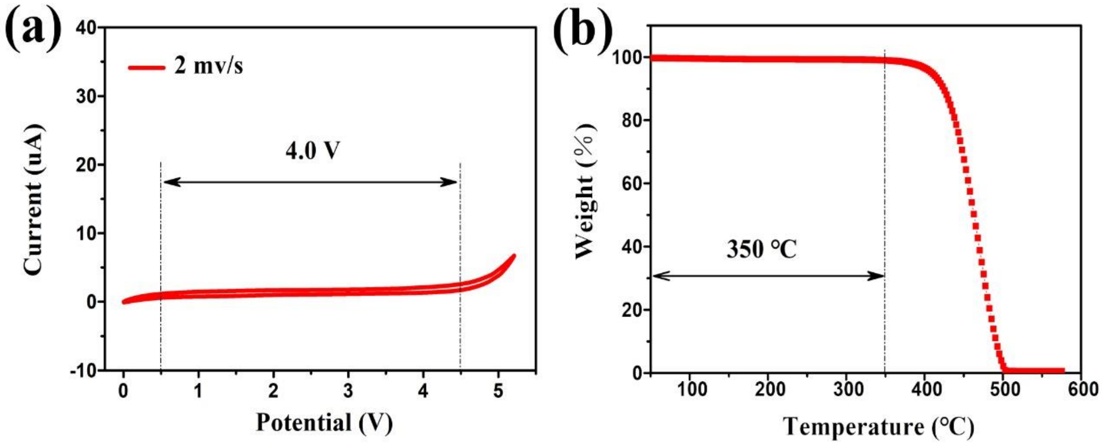

Figure S18. Electrochemical and thermal stability of $EMIMBF_4$ IL. (a) Cyclic voltammogram at a potential scan rate of 2 mV s^{-1} indicates that the $EMIMBF_4$ IL is stable at a broad potential window of about 4.0 V. (b) TGA thermogram under nitrogen atmosphere, with heating rate of 10 °C min^{-1}, demonstrates that the

$EMIMBF_4$ IL has excellent thermal stability.

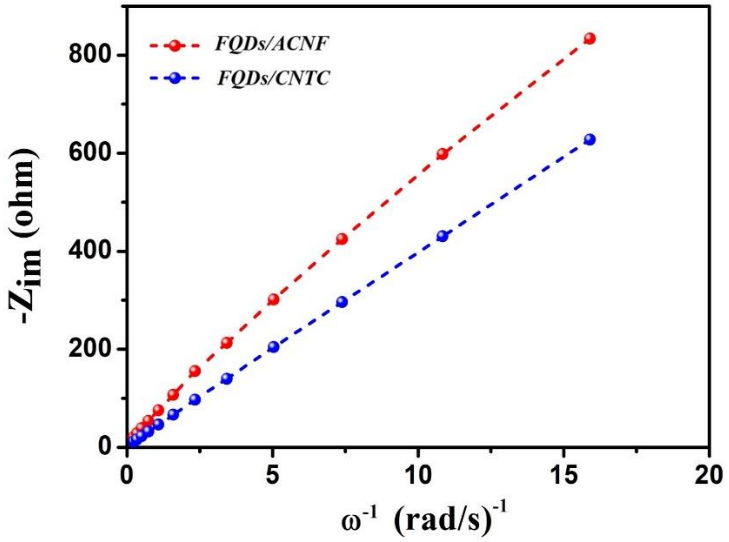

Figure S19. Plots of $-Z_{im}$ vs $1/\omega$ of FQDs/ACNF and FQDs/CNTC film electrodes in the $EMIMBF_4$ IL electrolyte.

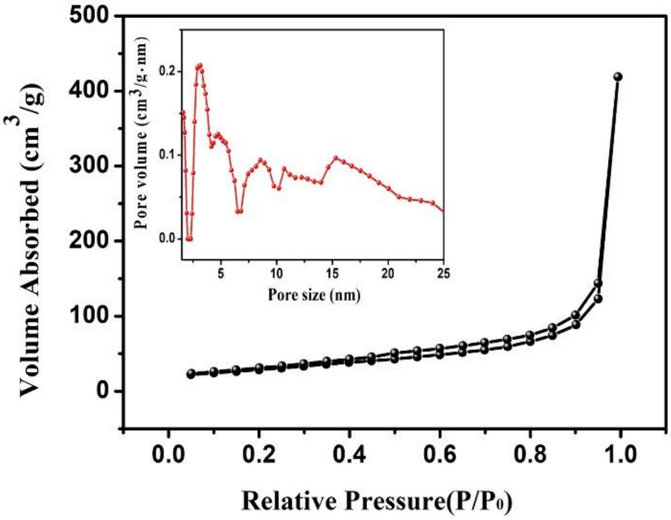

Figure S20. Nitrogen adsorption/desorption isotherm and pore size distribution curve of FQDs/CNTC film.

Nitrogen adsorption-desorption analysis reveals a specific surface area about 84.3 $m^2\ g^{-1}$ of the FQDs/CNTC film. Pore size distribution confirms abundant mesoporous structure of the FQDs/CNTC film.

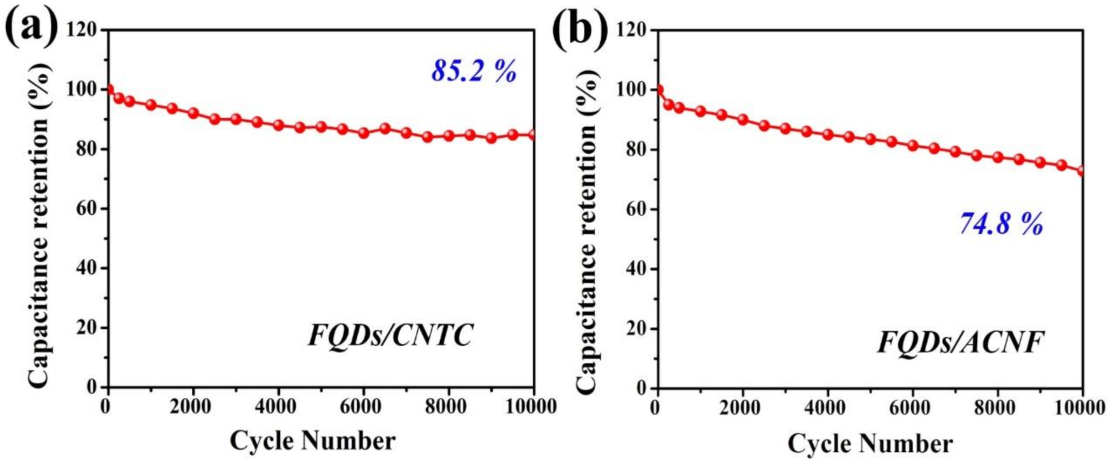

Figure S21. Comparison of cycle performance of the (a) FQDs/CNTC and (b) FQDs/ACNF film electrodes in the $EMIMBF_4$ IL electrolyte at a current density of 4 A cm^{-3}.

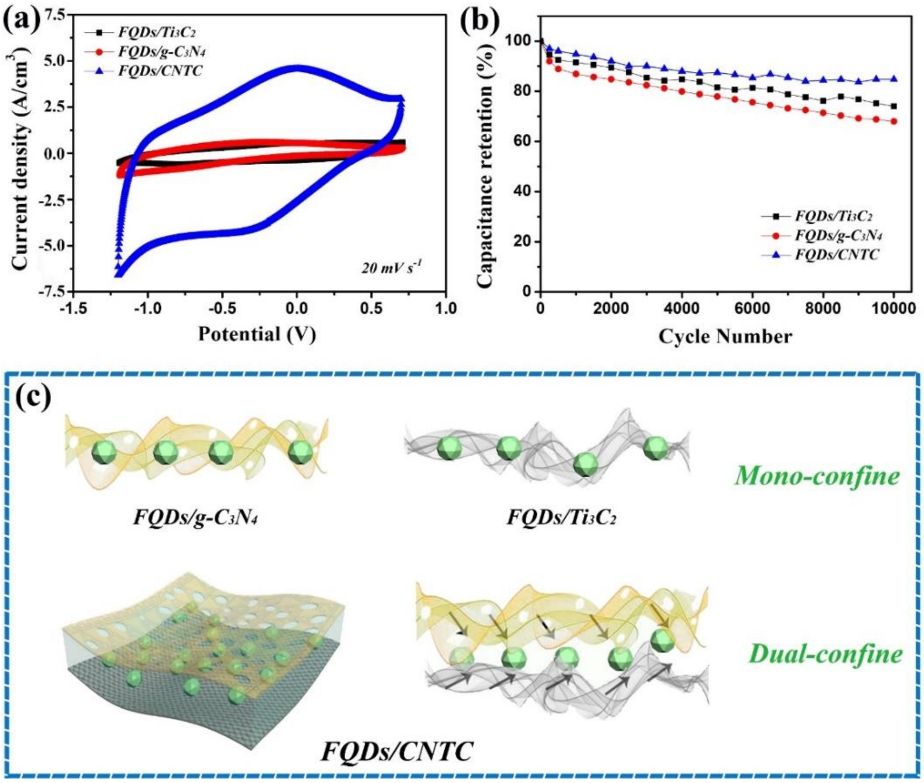

Figure S22. (a) CV curves and (b) cycle performances and (c) corresponding schematic diagrams of FQDs/g-C_3N_4, FQDs/Ti_3C_2 and FQDs/CNTC film electrodes in the $EMIMBF_4$ IL electrolyte.

***Preparation of FQDs/g-C_3N_4 and FQDs/Ti_3C_2 film electrodes*:** 40 mg porous g-C_3N_4 or 60 mg Ti_3C_2 powder was added to 50 mL ethanol with sonication for 5 min. Then, 1 mmol $FeCl_3 \cdot 6H_2O$ and 3 mmol NH_4HCO_3 were dissolved separately in above

solution with stirring for 10 h. The resultant FQDs/g-C_3N_4 or FQDs/Ti_3C_2 was collected via centrifugation, washed and dried. Different form FQDs/CNTC with good film forming ability, FQDs/g-C_3N_4 and FQDs/Ti_3C_2 are easily disintegrated after vacuum filtration, which could not directly act as the binder-free and flexible film electrode. For comparison, we prepared FQDs/g-C_3N_4 and FQDs/Ti_3C_2 film electrodes by common coating method. The mixture of FQDs/g-C_3N_4 or FQDs/Ti_3C_2 powder (85 wt %), acetylene black (5 wt %), and PTFE (10 wt %) was uniformly coated onto the surface of carbon film (thickness ~ 60 μm), followed by a drying-solidifying process in the oven to form FQDs/g-C_3N_4 and FQDs/Ti_3C_2 film electrodes.

As shown in Figure S22 (a)**,** the FQDs/CNTC film electrode displays significantly higher specific capacitance than that of the FQDs/g-C_3N_4 and FQDs/Ti_3C_2 film electrode, which is proved by its larger CV area. In general, the employment of inactive components including conductive additives and current collectors of the FQDs/g-C_3N_4 and FQDs/Ti_3C_2 film electrode makes the SCs too heavy and rigid to meet the practical requirements for portable devices. Furthermore, the introduction of insulating binder blocks the diffusion channels of charge transport and compromises the electrochemical performance. Besides, FQDs/CNTC film electrode shows good cycle performance, which is better than FQDs/g-C_3N_4 and FQDs/Ti_3C_2 film electrodes (Figure S22 (b)). This is due to the dual-confinement of FQDs in the FQDs/CNTC film electrode, providing a suitable nanospace to effectively inhibits the aggregation and dissolution of FQDs during the cycling processes (Figure S22 (c)).

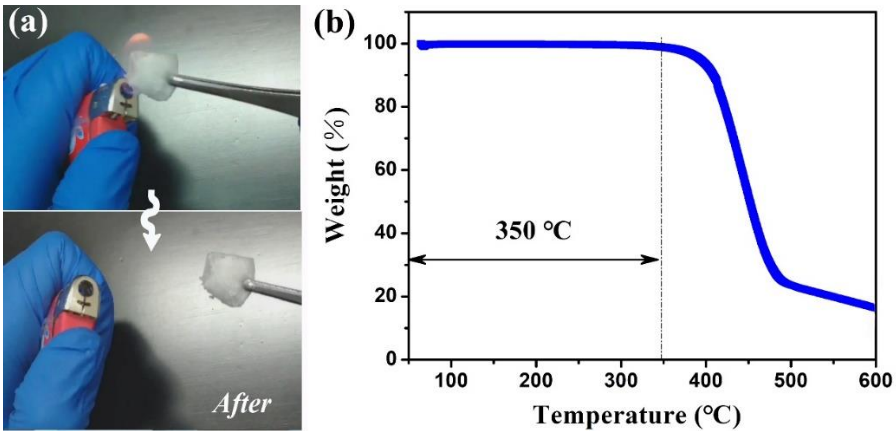

Figure S23. (a) Flammability test and (b) TGA curve of P(VDF-HFP)-$EMIMBF_4$ ionogel electrolyte.

As shown in Figure S23 (a), the P(VDF-HFP)-EMIMBF$_4$ ionogel electrolyte is nonflammable, thus providing high security and reliability for practical application in flexible electronics. Moreover, as seen from the TGA curve in Figure S23 (b), the ionogel electrolyte exhibits extremely high thermal stability above 350 °C, much better than that of aqueous gel electrolyte (< 100 °C). According to the four-probe method, the conductivity of P(VDF-HFP)-EMIMBF$_4$ ionogel electrolyte is measured to be 9.2 mS cm^{-1}, which is close to that of EMIMBF$_4$ IL electrolyte (13.4 mS cm^{-1}).

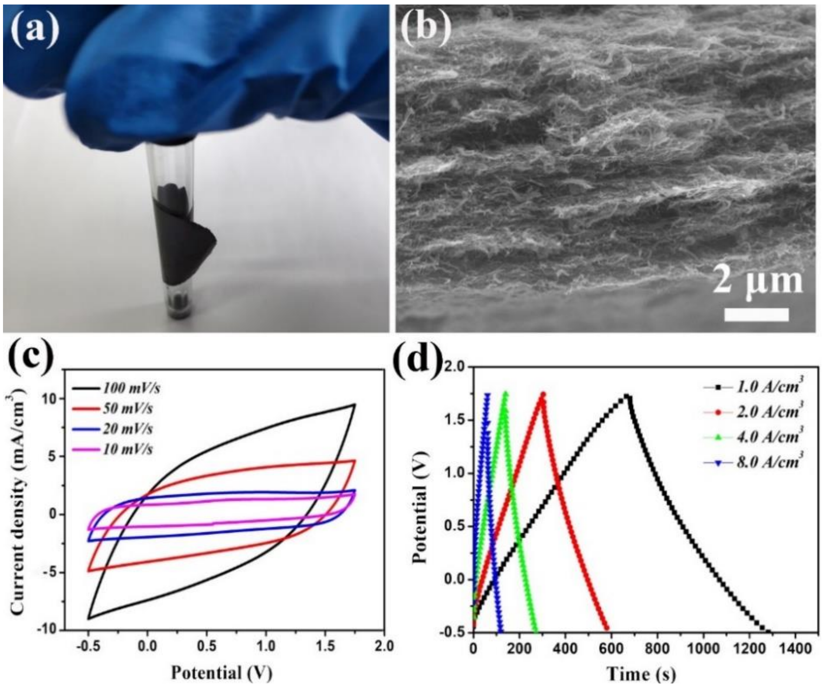

Figure S24. (a) Digital photo and (b) Cross-section FESEM image of CNTs/RGO film. (c) CV curves and (c) GCD curves of CNTs/RGO film electrode in the EMIMBF$_4$ IL electrolyte.

Raw carbon nanotubes (CNTs) were purified by calcination at 500 °C and washed repeatedly with HCl to eliminate impurities, and treated by a modified Hummers method with one-third amount of KMnO$_4$ according to the previous reports,[20, 21] which was then dispersed into deionized water to form homogeneous CNTs solution. Graphene oxide (GO) was synthesized using natural graphite (Alfa Aesar, 325 mesh) by a modified Hummer's method.[22] After that, the CNTs aqueous dispersion was mixed with the GO solution and then stirred continuously for 20 h, in which the mass ratio of CNTs and GO was about 40:60. Finally, the mixture solution was vacuum-filtrated through a filter membrane (0.22 μm pore size), and peeled from the

membrane, followed by heat-treatment at 200 °C for 2 h in N_2 to obtain CNTs/RGO film.

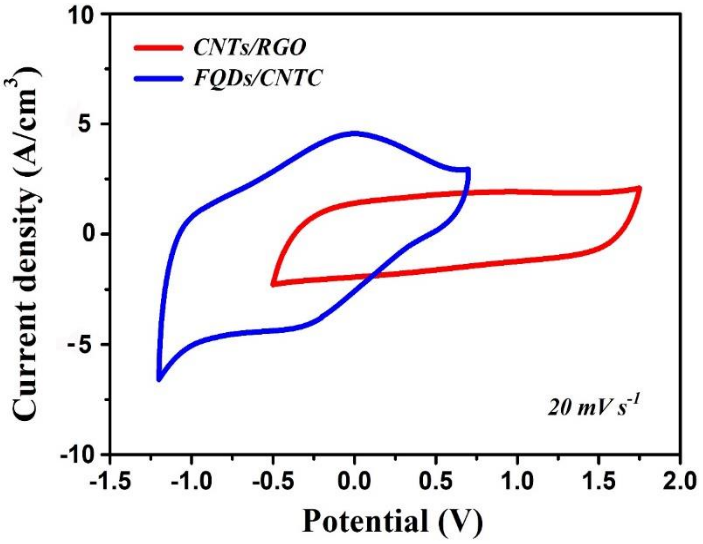

Figure S25. CV curves of individual FQDs/CNTC film and CNTs/RGO film electrodes in the $EMIMBF_4$ IL electrolyte at a scan rate of 20 mV s^{-1}.

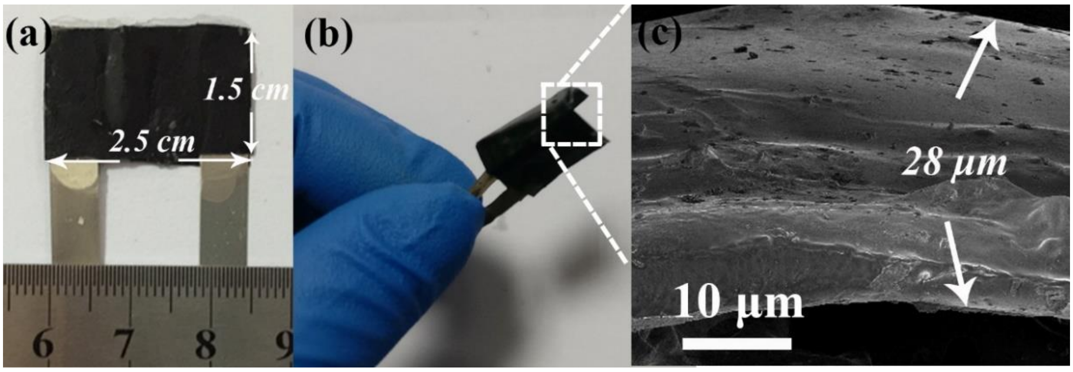

Figure S26. Digital photos of the FSC under (a) straight and (b) bending states. (c) Cross-section FESEM image of the FSC.

As shown in Figure S26, the thickness of FSC is about 28 μm, while the length and width are 2.5 cm and 1.5 cm, respectively, so the total volume of the device is about 0.0105 cm^3.

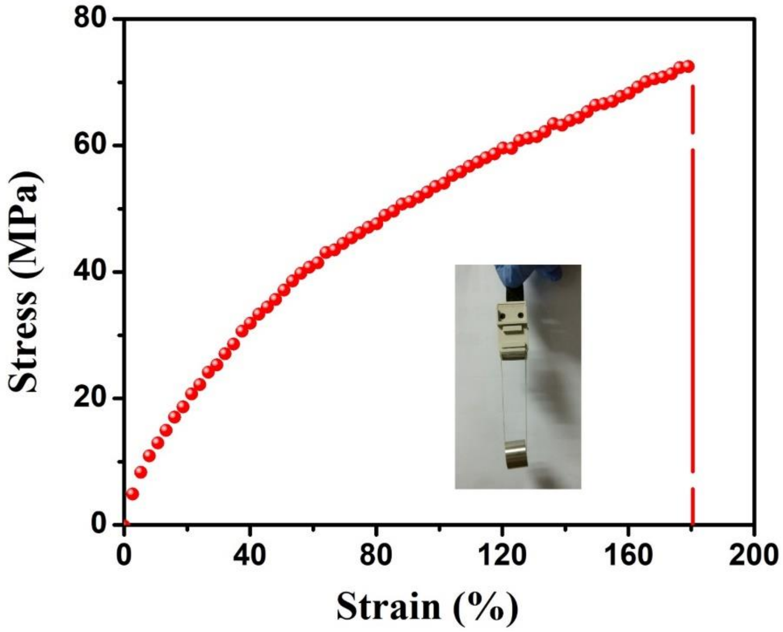

Figure S27. Typical stress–strain curve of the FSC. Inset is the digital photo of FSC with high strength (about 73.68 MPa).

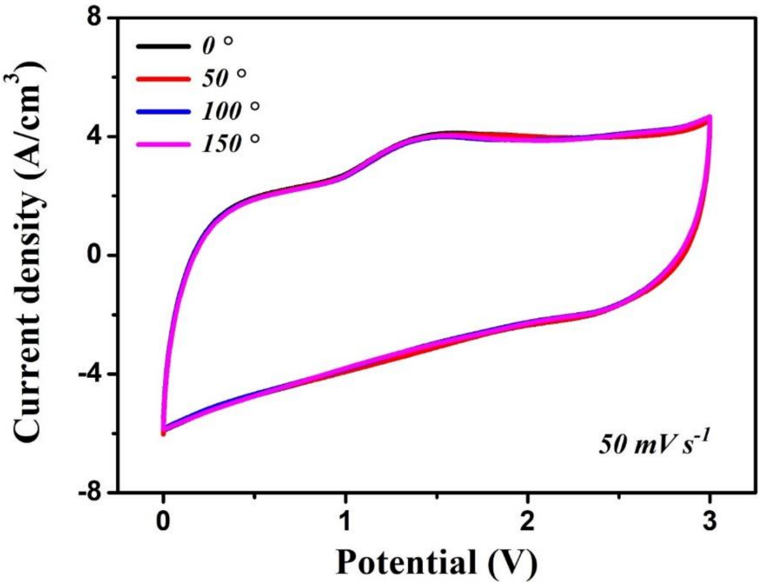

Figure S28. CV curves of the FSC under different mechanical deformation.

CV curves of the device have negligible changes under various conditions. It can maintain nearly 100% of its initial capacitance upon bending at different angles,

indicating high electrochemical reliability under harsh mechanical conditions.

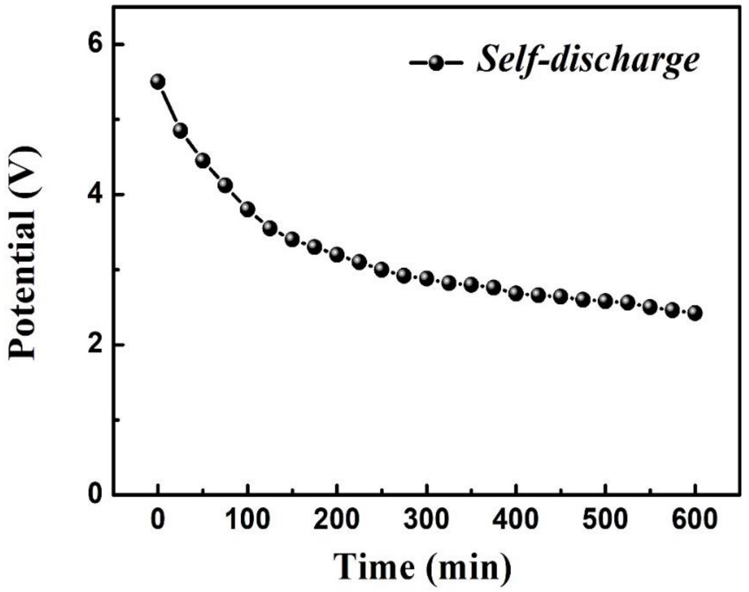

Figure S29. Self-discharge curves of four FSCs in a serial and parallel arrangement after harvesting the sustainable energy.

Table S1 The calculated value of liquid electrode uptake (*EU*) and porosity (*P*) of film electrode according to the actual weights (W_w and W_d) of EMIMBF$_4$ IL-soaked film and dry film.

Film electrode	W_d (mg)	W_w (mg)*	V(cm^3)	EU (%)	P (%)
FQDs/ACNF	8.32	10.53	0.0038	26.56	44.74
FQDs/CNTC	8.05	11.68	0.0030	45.09	93.07

* W_w is the weight of film electrode fully wetting with IL electrolyte for 1 h.

We have carefully calculated liquid electrode uptake (*EU*) and porosity (*P*) for both the FQDs/ACNF and FQDs/CNTC film electrodes in EMIMBF$_4$ IL electrolyte according to the following equations:

$EU(\%) = [(W_w - W_d)/W_d] \times 100\%$

$P\ (\%) = [(W_w - W_d)/(\rho \cdot V)] \times 100\%$

where W_w and W_d are the weight of IL-soaked film and dry film, respectively, ρ (~1.30 g cm^{-3}) is the density of $EMIMBF_4$ IL, and V is the geometric volume of the film. As shown in Table S1, the calculated *EU* and *P* of FQDs/CNTC film are higher than those of FQDs/ACNF film, further indicating the superior wettability of FQDs/CNTC film in $EMIMBF_4$ IL electrolyte.

Table S2. Energy densities of previously reported state-of-the-art FSCs based on film electrodes comparison with our result.

Film Electrode	Film-forming method	Volumetric energy density (mWh cm^{-3})	Ref.
**PEDOT film**	Spin coating	1.77	[23]
**Porous graphene film**	Sacrificial template	2.65	[24]
**Mn_3O_4/graphene film**	Vacuum filtration	5.50	[25]
**NiCo-LDH/graphene film**	Blade coating	5.10	[26]
**MXene/graphene film**	Vacuum filtration	3.40	[27]
**graphene/CNTs film**	Vacuum filtration	1.70	[28]
**Black phosphorus/CNTs film**	Vacuum filtration	5.71	[29]
**$NiCo_2O_4$/CNTs film**	Chemical vapor deposition	1.17	[30]
**$Na_2W_4O_{13}$/CNTs film**	Dip coating	3.83	[31]
**V-doped MnO_2/CNTs film**	Vacuum filtration	4.98	[32]
**Grpahene/Ti_3C_2 film**	Vacuum filtration	63.0	[33]
**PEDOT:PSS/ Ti_3C_2 film**	Vacuum filtration	23.0	[34]
**MnO_x/Ti_3C_2 film**	Vacuum filtration	13.64	[35]
**MnO_2/Ti_3C_2 film**	Vacuum filtration	56.94	[36]
**FQDs/CNTC film**	Vacuum filtration	77.12	This work

References

[1] L. Q. Yang, J. F. Huang, L. Shi, L. Y. Cao, Q. Yu, Y. N. Jie, J. Fei, H. B. Ouyang, J. H. Ye, *Appl. Catal., B.* **2017**, *204*, 335.

[2] H. Xu, J. J. Yi, X. J. She, Q. Liu, L. Song, S. M. Chen, Y. C. Yang, Y. H. Song, R. Vajtai, J. Lou, H. M. Li, S. Q. Yuan, J. J. Wu, P. M. Ajayan, *Appl. Catal., B* **2018**, *220*, 379.

[3] P. Niu, L. L. Zhang, G. Liu, H. M. Cheng, *Adv. Funct. Mater.* **2012**, *22*, 4763.

[4] Y. H. Song, X. J. She, J. J. Yi, Z. Mo, L. Liu, H. Xu, H. M. Li, *Phys. Status Solidi* **2017**, *5*, 1600704.

[5] X. J. She, J. J. Wu, J. Zhong, H. Xu, Y. C. Yang, R. Vajtai, J. Lou, Y. Liu, D. L. Du, H. M. Li, P. M. Ajayan, *Nano Energy* **2016**, *27*, 138.

[6] Y. T. Xiao, G. H. Tian, W. Li, Y. Xie, B. J. Jiang, C. G. Tian, D. Y. Zhao, H. G. Fu, *J. Am. Chem. Soc.* **2019**, *141*, 2508.

[7] F. Guo, W. L. Shi, M. H. Li, Y. Shi, H. B. Wen, *Sep. Purif. Technol.* **2019**, *210*, 608.

[8] A. Noda, K. Hayamizu, M. Watanabe, *J. Phys. Chem. B* **2001**, *105*, 4603.

[9] R. Fortunato, L. C. Branco, C. A. M. Afonso, J. Benavente, J. G. Crespo, *J. Membr. Sci.* **2006**, *270*, 42.

[10] V. Lockett, R. Sedev, J. Ralston, M. Horne, T. Rodopoulos, *J. Phys. Chem. C* **2008**, *112*, 7486.

[11] M. V. Fedorov, N. Georgi, A. A. Kornyshev, *Electrochem. Commun.* **2010**, *12*, 296.

[12] S. Jo, S. W. Park, Y. Shim, Y. Jung, *Electrochim. Acta* **2017**, *247*, 634.

[13] J. Vatamanu, O. Borodin, G. D. Smith, *J. Am. Chem. Soc.* **2010**, *132*, 14825.

[14] J. F. Li, P. H. Q. Pham, W. W. Zhou, T. D. Pham, P. J. Burke, *ACS Nano* **2018**, *12*, 9763.

[15] C. R. Mariappan, T. P. Heins, B. Roling, *Solid State Ionics* **2010**, *181*, 859.

[16] P. S. Gil, S. J. Jorgenson, A. R. Riet, D. J. Lacks, *J. Phys. Chem. C* **2018**, *122*, 27462.

[17] M. J. Shi, S. Z. Kou, X. B. Yan, *Chemsuschem* **2014**, *7*, 3053.

[18] H. Y. Che, S. L. Chen, Y. Y. Xie, H. Wang, K. Amine, X. Z. Liao, Z. F. Ma, *Energy Environ. Sci.* **2017**, *10*, 1075.

[19] W. Lu, K. Henry, C. Turchi, J. Pellegrino, *J. Electrochem. Soc.* **2008**, *155*, A361.

[20] T. Y. Ma, S. Dai, M. Jaroniec, S. Z. Qiao, *Angew. Chem., Int. Ed.* **2014**, *53*, 7281.

[21] Y. Y. Liang, H. L. Wang, P. Diao, W. Chang, G. S. Hong, Y. G. Li, M. Gong, L. M. Xie, J. G. Zhou, J. Wang, T. Z. Regier, F. Wei, H. J. Dai, *J. Am. Chem. Soc.* **2012**, *134*, 15849.

[22] L. Wang, M. J. Shi, C. Yang, Y. C. Liu, J. T. Jiang, K. Dai, Z. H. Guo, C. Yan, *J. Alloys Compd.* **2019**, *804*, 243.

[23] Z. F. Li, G. Q. Ma, R. Ge, F. Qin, X. Y. Dong, W. Meng, T. F. Liu, J. H. Tong, F. Y. Jiang, Y. F. Zhou, K. Li, X. Min, K. F. Huo, Y. H. Zhou, *Angew. Chem., Int. Ed.* **2016**, *55*, 979.

[24] K. Q. Qin, J. L. Kang, J. J. Li, E. Z. Liu, C. S. Shi, Z. J. Zhang, X. X. Zhang, N. Q. Zhao, *Nano Energy* **2016**, *24*, 158.

[25] Y. T. Hu, C. Guan, G. X. Feng, Q. Q. Ke, X. L. Huang, J. Wang, *Adv. Funct. Mater.* **2015**, *25*, 7291.

[26] D. D. Jia, D. G. Jiang, Y. W. Zheng, H. Tan, X. Y. Cao, F. Liu, L. J. Yue, Y. Y. Sun, J. Q. Liu, *Nanoscale* **2019**, *11*, 2812.

[27] H. Y. Li, Y. Hou, F. X. Wang, M. R. Lohe, X. D. Zhuang, L. Niu, X. L. Feng, *Adv. Energy Mater.* **2017**, *7*, 6.

[28] X. B. Zang, R. J. Zhang, Z. Zhen, W. H. Lai, C. Yang, F. Y. Kang, H. W. Zhu, *Nano Energy* **2017**, *40*, 224.

[29] B. C. Yang, C. X. Hao, F. S. Wen, B. C. Wang, C. P. Mu, J. Y. Xiang, L. Li, B. Xu, Z. S. Zhao, Z. Y. Liu, Y. J. Tian, *ACS Appl. Mater. Interfaces* **2017**, *9*, 44478.

[30] Y. J. Zheng, Z. Q. Lin, W. J. Chen, B. H. Liang, H. W. Du, R. L. Yang, X. F. He, Z. K. Tang, X. C. Gui, *J. Mater. Chem. A* **2017**, *5*, 5886.

[31] Z. M. Hu, X. Xiao, H. Y. Jin, T. Q. Li, M. Chen, Z. Liang, Z. F. Guo, J. Li, J. Wan, L. Huang, Y. R. Zhang, G. Feng, J. Zhou, *Nat. Commun.* **2017**, *8*, 9.

[32] Z. M. Hu, X. Xiao, L. Huang, C. Chen, T. Q. Li, T. C. Su, X. F. Cheng, L. Miao, Y. R. Zhang, J. Zhou, *Nanoscale* **2015**, *7*, 16094.

[33] S. K. Xu, G. D. Wei, J. Z. Li, W. Han, Y. Gogotsi, *J. Mater. Chem. A* **2017**, *5*, 17442.

[34] L. Li, N. Zhang, M. Y. Zhang, X. T. Zhang, Z. G. Zhang, *Dalton. Trans.* **2019**, *48*, 1747.

[35] Y. P. Tian, C. H. Yang, W. X. Que, X. B. Liu, X. T. Yin, L. B. Kong, *J. Power Sources* **2017**, *359*, 332.

[36] J. Zhou, J. L. Yu, L. D. Shi, Z. Wang, H. C. Liu, B. Yang, C. H. Li, C. Z. Zhu, J. Xu, *Small* **2018**, *14*, 1803786.
